# Interrater agreement of contouring of the neurovascular bundles and internal pudendal arteries in neurovascular-sparing magnetic resonance-guided radiotherapy for localized prostate cancer

**DOI:** 10.1016/j.ctro.2021.11.005

**Published:** 2021-11-14

**Authors:** F.R. Teunissen, R.C. Wortel, F.J. Wessels, A. Claes, S.M.G. van de Pol, M.J.A. Rasing, R.P. Meijer, H.H.E. van Melick, J.C.J. de Boer, H.M. Verkooijen, J.R.N. van der Voort van Zyp

**Affiliations:** a Department of Radiation Oncology, University Medical Center Utrecht, Utrecht, the Netherlands; b Department of Oncological Urology, University Medical Center Utrecht, Utrecht, the Netherlands; c Department of Radiology, University Medical Center Utrecht, Utrecht, the Netherlands; d Department of Urology, St. Antonius Hospital, Nieuwegein, Utrecht, the Netherlands; e Imaging and Oncology Division, University Medical Center Utrecht, Utrecht, the Netherlands; f Utrecht University, Utrecht, the Netherlands

**Keywords:** CC, corpus cavernosum, CT, computed tomography, DSC, Dice similarity coefficient, EBRT, external beam radiation therapy, GRRAS, Guidelines for Reporting Reliability and Agreement Studies, Gy, gray, IPA, internal pudendal artery, IQR, interquartile range, MRgRT, magnetic resonance-guided adaptive radiotherapy, MRI, magnetic resonance imaging, NCCN, National Comprehensive Cancer Network, NVB, neurovascular bundle, OAR, organs at risk, PB, penile bulb, PCa, prostate cancer, PTV, planning target volume, Localized prostate cancer (PCa), Magnetic resonance-guided radiotherapy (MRgRT), Interrater agreement, Neurovascular bundle (NVB), Internal pudendal artery (IPA), Neurovascular-sparing, Erectile function sparing

## Abstract

**Background and purpose:**

Radiation damage to neural and vascular tissue, such as the neurovascular bundles (NVBs) and internal pudendal arteries (IPAs), during radiotherapy for prostate cancer (PCa) may cause erectile dysfunction. Neurovascular-sparing magnetic resonance-guided adaptive radiotherapy (MRgRT) aims to preserve erectile function after treatment. However, the NVBs and IPAs are not routinely contoured in current radiotherapy practice. Before neurovascular-sparing MRgRT for PCa can be implemented, the interrater agreement of the contouring of the NVBs and IPAs on pre-treatment MRI needs to be assessed.

**Materials and methods:**

Four radiation oncologists independently contoured the prostate, NVB, and IPA in an unselected consecutive series of 15 PCa patients, on pre-treatment MRI. Dice similarity coefficients (DSCs) for pairwise interrater agreement of contours were calculated. Additionally, the DCS of a subset of the inferior half of the NVB contours (i.e. approximately prostate midgland to apex level) was calculated.

**Results:**

Median overall interrater DSC for the left and right NVB was 0.60 (IQR: 0.54 – 0.68) and 0.61 (IQR: 0.53 – 0.69) respectively and for the left and right IPA 0.59 (IQR: 0.53 – 0.64) and 0.59 (IQR: 0.52 – 0.64) respectively. Median overall interrater DSC for the inferior half of the left NVB was 0.67 (IQR: 0.58 – 0.74) and 0.67 (IQR: 0.61 – 0.71) for the right NVB.

**Conclusion:**

We found that the interrater agreement for the contouring of the NVB and IPA improved with enhancement of the MRI sequence as well as further training of the raters. The agreement was best in the subset of the inferior half of the NVB, where a good agreement is clinically most relevant for neurovascular-sparing MRgRT for PCa.

## Highlights

• Interrater DSC of the NVB was 0.60 and 0.61 for the left and right side respectively.
• Interrater DSC of the IPA was 0.59 for the left and right side.
• Agreement was best for the inferior half (i.e. prostate apex to midgland) of the NVB.
• Agreement improved with MRI optimization and rater training.

## Introduction

Erectile dysfunction is a common adverse effect of external beam radiation therapy (EBRT) for localized prostate cancer (PCa) [1]. The prostate is located adjacent to neural structures and in close proximity to vascular structures responsible for erectile function such as the neurovascular bundles (NVBs), the internal pudendal arteries (IPAs), the corpora cavernosa (CCs), and the penile bulb (PB) [2]. Radiation damage to these structures can lead to temporary or permanent decline of erectile function [3], [4], [5].

Implementation of neurovascular-sparing radiotherapy treatments has been impeded by the routine use of computed tomography (CT) imaging in radiotherapy treatment planning. Using CT imaging, CCs and PB can be identified sufficiently, and PB is often included as organ at risk (OAR) in conventional EBRT [6], [7]. However, other critical structures such as the NVBs and IPAs cannot be adequately identified on CT and are therefore not spared in conventional EBRT [2], [8].

The integration of Magnetic Resonance Imaging (MRI) has improved imaging and enables functional anatomy-based radiotherapy treatment planning [2]. Furthermore, the recent development of magnetic resonance-guided adaptive radiotherapy (MRgRT) has enabled real-time high-resolution MRI imaging during dose delivery and facilitates correction for both inter- and intra-fraction movement and tissue deformations [9]. Planning target volumes (PTV) can therefore be smaller as safety margins are reduced [10]. These improvements in treatment conformity using MRgRT facilitate neurovascular-sparing radiotherapy and could potentially improve sexual function outcomes in patients treated for localized PCa. As the NVBs and IPAs are not routinely contoured in current clinical radiotherapy practice, we aimed to assess the interrater agreement in contouring the NVBs and IPAs on pre-treatment MRI.

## Materials and methods

### Patient selection and treatment

For this study the guidelines for reporting reliability and agreement studies (GRRAS) recommendations were followed [11]. Included were patients with localized PCa (low to high risk according to the National Comprehensive Cancer Network [NCCN] risk classification) that received MRI guided external beam radiation therapy (MRgRT) in five fractions of 7.25 Gray (Gy) delivered during two and a half weeks on a 1.5 T MR-Linac system. All patients received a single 3.0 T planning MRI and 1.5 T pretreatment MRIs for daily plan adaptation prior to each fraction. Patients signed informed consent for sharing of their clinical data within the MOMENTUM study (NCT04075305), which was approved by our institutional review board [12].

### Contouring instructions and pilot study

First, a consensus meeting was held with two radiologists and four radiation oncologists all dedicated in uro-oncology to determine the location and set the anatomical boundaries for NVB and IPA on MR imaging. After consensus was reached, a contouring atlas was developed (Supplementary material). Subsequently, a pilot study was initiated in which four senior dedicated prostate radiation oncologists (25, 15, 10, 10 years of experience respectively) independently contoured the prostate, NVBs, and IPAs in an unselected series of five consecutive patients on a single clinical pretreatment 3.0 T T2-weighted MR scan. All raters contoured the structures individually and were blinded for the contours of the other raters. The in-house developed contouring software package Volumetool was used, which is also used for clinical contouring at our institution. All raters had access to the contouring atlas and were instructed to contour the NVB from at least the base of the seminal vesicles until the level of the urogenital diaphragm and the IPA from at least the level of the sacroiliac ligament until the level of the crus where it terminates into the common penile artery and the scrotal artery. Subsequently, per patient the four contours of the left and right NVB and left and right IPA were adjusted to run from the same superior to inferior level, based on the maximal superior to inferior distance overlap of the four contours, for every structure independently. Furthermore, a subset of the exact inferior half (i.e. approximately prostate midgland to apex level) of the NVB contours was generated, where the NVB generally is in closest proximity to the prostate and conflict between optimal dose coverage and sparing of neurovascular structures is greatest.

### Main study

An evaluation of the pilot study was added to the contouring atlas. Also, an optimized 3D TSE MR-sequence was developed to improve NVB and IPA visualization, which became our institution’s standard for daily contouring and replaced the previous T2-weighted sequence (Supplementary material). Four dedicated prostate radiation oncologists (15, 10, 5, 3 years of experience respectively), independently contoured the prostate, the left and right NVB and the left and right IPA in a new unselected series of 15 consecutive patients. Rater one and two also participated in the pilot study. All raters contoured the structures individually and were blinded for the contours of the other raters. Contouring was done on a single clinical pretreatment 1.5 T T2-weighted MR scan of the pelvis that was acquired on an MR-Linac. The software package Volumetool was used for contouring.

All raters had access to the contouring atlas and evaluation of the pilot study, and were instructed to contour the NVB from at least the base of the seminal vesicles until the level of the urogenital diaphragm and the IPA from at least the level of the sacroiliac ligament until the level of the crus where it terminates into the common penile artery and the scrotal artery. Subsequently, per patient the four contours of the left and right NVB and left and right IPA were adjusted to run from the same superior to inferior level and a subset of the exact inferior half of the NVB contours was generated.

### Statistical analysis

Interrater agreement was assessed by calculating the Dice similarity coefficient (DSC) of all possible rater pairs (i.e. four raters result in six rater pairs per patient) [13]. DSC = 0 indicates no spatial overlap, 0 < DSC < 1 indicates partial spatial overlap and DSC = 1 indicates complete spatial overlap between two contours. Complementary average surface distance and maximum surface distance (i.e. Hausdorff distance) between the contours of all rater pairs were calculated [14], [15]. Distances were calculated symmetrically and 3-dimensional. An average surface distance or Hausdorff distance of 0 indicates perfect overlap between two contours. Volume, DSC, average surface distance, and Hausdorff distance were calculated by analysis modules accompanying Volumetool. Distances are represented in mm and volumes in cc. Non-normally distributed data were presented as median with interquartile range (IQR).

## Results:

### Pilot study

The pilot study included five patients which were each contoured by four raters. The mean age of the patients was 68 years old (range: 57 years − 80 years), one had low-risk, and four had intermediate-risk PCa. The median overall contoured volume was 3.54 cc (2.74 cc – 4.16 cc) for the left and 3.74 cc (2.88 cc – 4.87 cc) for the right NVB, and 2.83 cc (2.03 cc – 4.32 cc) for the left and 2.96 cc (1.86 cc – 3.96 cc) for the right IPA (Table 1).

**Table 1:** Interrater agreement outcomes of contouring of the prostate, IPAs, and NVBs.

		Prostate	IPA left	IPA right	NVB left	NVB right	NVB inferior half left	NVB inferior half right
		Median (IQR)	Median (IQR)	Median (IQR)	Median (IQR)	Median (IQR)	Median (IQR)	Median (IQR)
Pilot study (5 patients)	Overal volume (cc) n = 20	27.87 (23.80 – 31.93)	2.83 (2.03 – 4.32)	2.96 (1.86 – 3.96)	3.54 (2.74 – 4.16)	3.74 (2.88 – 4.87)	1.21 (0.93 – 1.39)	1.44 (1.11 – 1.72)
	Overall distance between superior and inferior border (mm) n = 5	NA	42.00 (42.00 – 46.00)	44.00 (38.00 – 46.00)	34.00 (32.00 – 38.00)	32.00 (32.00 – 40.00)	18.00 (16.00 – 20.00)	16.00 (16.00 – 20.00)
	Overall Dice similarity coefficent n = 30*	0.84 (0.82 – 0.87)	0.57 (0.47 – 0.63)	0.46 (0.29 – 0.56)	0.42 (0.32 – 0.52)	0.51 (0.40 – 0.59)	0.50 (0.35 – 0.57)	0.50 (0.41 – 0.60)
	Overall average surface distance (mm) n = 30*	1.81 (1.38 – 2.02)	1.36 (1.12 – 2.38)	1.91 (1.28 – 3.39)	2.64 (1.77 – 3.36)	1.89 (1.59 – 2.38)	1.48 (1.16 – 1.97)	1.34 (1.20 – 1.84)
	Overall Hausdorff distance (mm) n = 30*	7.17 (5.98 – 8.02)	8.60 (6.61 – 12.58)	11.98 (7.25 – 19.23)	11.89 (9.48 – 17.02)	9.16 (8.07 – 11.35)	6.67 (5.01 – 8.10)	6.44 (5.38–8.37)
Main study (15 patients)	Overall volume (cc) n = 60	47.38 (34.45–56.84)	2.26 (1.77–2.95)	2.27 (1.66–2.97)	6.18 (5.22–8.31)	7.19 (5.83–9.08)	2.09 (1.51–2.85)	2.08 (1.54–2.77)
	Overall distance between superior and inferior border (mm) n = 15	NA	42.00 (34.00 – 42.00)	38.00 (34.00 – 47.00)	46.00 (43.00 – 51.00)	48.00 (43.00 – 51.00)	24.00 (22.00 – 26.00)	24.00 (22.00 – 26.00)
	Overall Dice similarity coefficient n = 90**	0.91 (0.89–0.92)	0.59 (0.53–0.64)	0.59 (0.52–0.64)	0.60 (0.54–0.68)	0.61 (0.53–0.69)	0.67 (0.58–0.74)	0.67 (0.61–0.71)
	Overall average surface distance (mm) n = 90**	1.24 (1.09 – 1.55)	1.18 (1.05 – 1.63)	1.24 (1.01 – 1.49)	1.96 (1.59 – 2.31)	1.86 (1.53 – 2.52)	1.10 (0.89 – 1.42)	1.21 (1.04 – 1.51)
	Overall Hausdorff distance (mm) n = 90**	5.99 (5.38–7.80)	8.24 (6.75 – 10.37)	7.84 (6.14 – 9.47)	12.13 (9.36 – 15.10)	11.78 (9.52–14.22)	6.39 (5.12 – 9.04)	7.84 (5.78 – 10.28)

Abbreviations: NVB = neurovascular bundle; IPA = internal pudendal artery; DSC = Dice similarity coefficient; IQR = interquartile range; NA = not applicable

*4 raters result in 6 rater comparisons (6 × 5 patients = 30)

**4 raters result in 6 rater comparisons (6 × 15 patients = 90)

Median overall interrater DSC (agreement) for the pilot study was 0.42 (IQR: 0.32 – 0.52) for the left NVB and 0.51 (IQR: 0.40 – 0.59) for the right NVB, and 0.57 (IQR: 0.47 – 0.63) for the left and 0.46 (IQR: 0.29 – 0.56) for the right IPA (Table 1). For the inferior half of the NVBs the median overall interrater DSC was 0.50 (IQR: 0.35 – 0.57) and 0.50 (IQR: 0.41 – 0.60) for the left and right side respectively.

Median overall average surface distance was 2.64 mm (IQR: 1.77 mm – 3.36 mm) and 1.89 mm (IQR: 1.59 mm – 2.38 mm) for the left and right NVB respectively, and 1.36 mm (IQR: 1.12 mm – 2.38 mm) and 1.91 mm (IQR: 1.28 mm – 3.39 mm) for the left and right IPA respectively (Table 1).

### Main study

For the main study, 15 patients were included which were each contoured by four raters. Mean age of the patients was 70 years old (range: 59 years – 79 years), one had low-risk, 11 had intermediate-risk, and two had high-risk PCa. The median overall contoured volume was 6.18 cc (5.22 cc – 8.31 cc) for the left and 7.19 cc (5.83 cc – 9.08 cc) for the right NVB, and 2.26 cc (1.77 cc – 2.95 cc) for the left and 2.27 cc (1.66 cc – 2.97 cc) for the right IPA (Table 1).

**Fig. 1:**
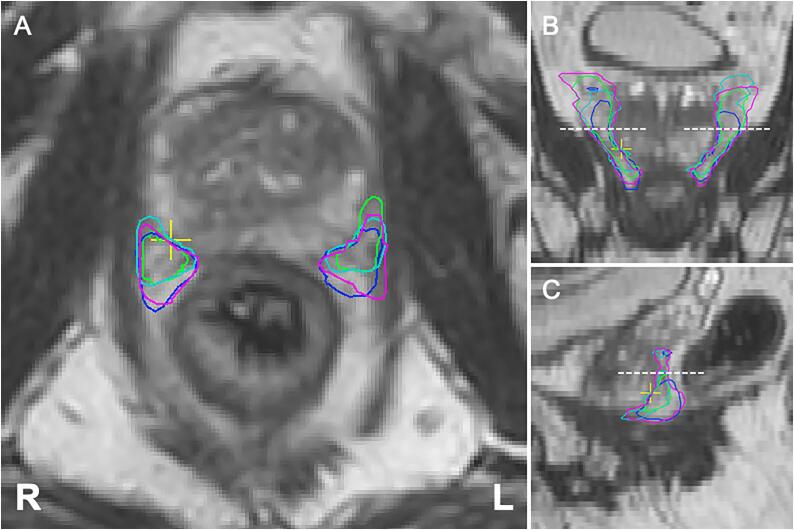
Representative case of contours of the neurovascular bundles (prostate apex level) by four raters.

**Fig. 2:**
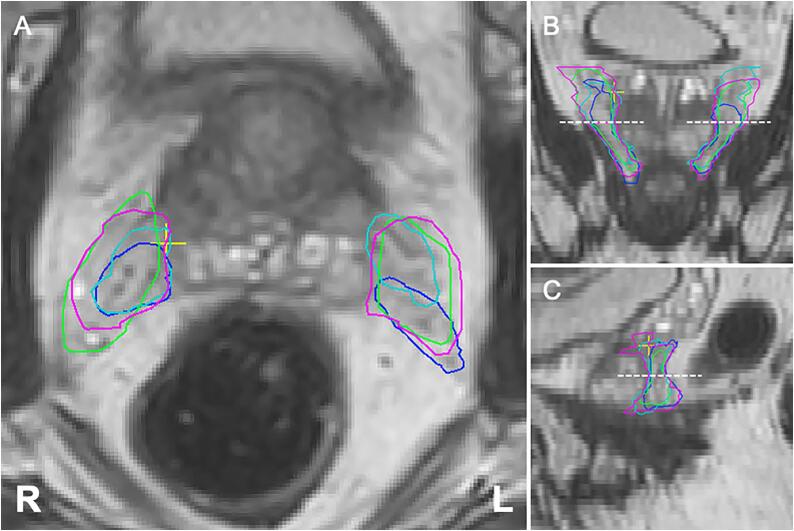
Representative case of contours of the neurovascular bundles (prostate base level) by four raters.

**Fig. 3:**
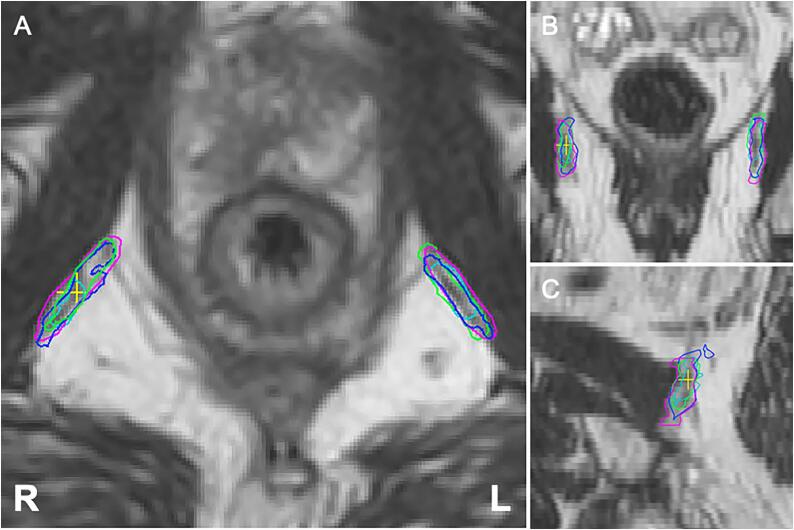
Representative case of contours of the internal pudendal arteries by four raters.

Median overall interrater DSC was 0.60 (IQR: 0.54 – 0.68) and 0.61 (IQR: 0.53 – 0.69) for the left and right NVBs respectively, and 0.59 (IQR: 0.53 – 0.64) and 0.59 (IQR: 0.52 – 0.64) for the left and right IPAs respectively (Table 1; Fig. 1, Fig. 2, Fig. 3). Assessment of the agreement of the inferior half of the NVBs resulted in a median overall interrater DSC of 0.67 (IQR: 0.58 – 0.74) for the left side and 0.67 (IQR: 0.61 – 0.71) for the right side.

Median overall average surface distance was 1.18 mm (IQR: 1.05 mm – 1.63 mm) and 1.24 mm (IQR:1.01 mm – 1.49 mm) for the left and right IPA respectively, and 1.96 mm (IQR: 1.59 mm – 2.31 mm) and 1.86 mm (IQR: 1.53 mm – 2.52 mm) for the left and right NVB respectively (Table 1).

## Discussion

This study is the first to assess the interrater agreement of both the NVB and IPA on MRI for MRgRT. Assessment of the interrater agreement of the contours of the NVB and the IPA on pre-treatment MRI resulted in a median overall DSC of 0.60 (IQR: 0.54 – 0.68) and 0.61 (IQR: 0.53 – 0.69) for the left and right NVB respectively and 0.59 (IQR: 0.53 – 0.64) for the left and 0.59 (IQR: 0.52 – 0.64) for the right IPA.

In literature, a DSC of > 0.70 is often deemed as excellent agreement, referring to a study of Zijdenbos et al [16]. However, in that study the agreement between a semiautomatic multispectral segmentation technique and manually contoured white matter lesions in the brain was assessed. This is by no means directly comparable with the field of oncology. For example: a DSC of 0.70 between raters can be considered low for the contouring of a tumor, where all tumor tissue should be treated, but may be excellent for a structure-to-spare that conventionally is not spared at all, which should be taken into account when interpreting the DSC.

Although DSC is the most frequently used metric for contour agreement, it is advised to accompany the DSC with additional metrics such as the average surface distance and Hausdorff distance between contours, to put the DSC in perspective [14], [15]. The DSC is comprehensible and works well as a crude measure of agreement, but has a lower sensitivity for fine details such as complex structure boundaries. Furthermore, a similar difference in terms of distance between two contours will result in a lower DSC between smaller volume contours as between larger volume contours. Therefore, volumetric overlap and distance metrics are generally not highly correlated and therefore should be used complementary. Taking the prostate in our study as a reference, the DSC of the IPAs and NVBs are substantially lower, however, average surface distances and Hausdorff distances are much more similar, especially for the inferior part of the NVB in comparison with the prostate (Table 1).

The few studies that have assessed the contouring agreement of the NVB, reported very different results. Cassidy et al. reported a mean DSC of 0.72 (standard deviation [SD]: 0.07) for the agreement of five radiation oncologists with a single “golden standard” contour of a radiologist in 10 cases [17]. However, the T2 3 T MRI scans were pre-selected to only contain patients with a favorable and consistent NVB anatomy. Also, a better overall DSC is expected when all raters are compared with a single “golden standard” opposed to pairwise rater comparison. These factors may have led to an overestimation of the agreement. On the contrary, Roach et al. reported a mean DSC of 0.16 (SD: 0.17) for the left and 0.15 (SD: 0.15) for the right NVB for the interrater agreement of 13 raters contouring five cases, showing almost no agreement between raters [18]. They contoured nine different structures on small field‐of‐view T2‐weighted MRI within the study, without specified training in contouring the NVBs, which could have led to a lower accuracy, resulting in a very low interrater DSC of the NVB contours.

To our knowledge, no studies on agreement of the contouring the IPA on MRI have been reported. However, a number of studies have shown the feasibility and potential of IPA sparing radiotherapy [2], [8], [19]. Spratt et al. conducted a single arm study sparing the CC and IPA during conventional EBRT for PCa in 135 patients and showed that 88% of patients were still sexually active with or without the use of aids five years after treatment, while maintaining tumor control [19]. To date, this is the only study addressing the effect of sparing of the IPA on erectile function preservation.

We consider the agreement for the NVB and IPA in our study to be acceptable for the implementation of neurovascular-sparing MRgRT, taking into account the DSC together with the average surface distance and Hausdorff distance. Moreover, subanalysis of the contours of the inferior part of the NVB showed a better DSC between raters (median overall DSC NVB left: 0.67 [IQR: 0.58 – 0.74], right: 0.67 [IQR: 0.61 – 0.71]) compared to the total contoured NVB and showed a relatively low median overall average surface distance of 1.10 mm (IQR: 0.89 mm – 1.42 mm) for the left and 1.21 mm (IQR: 1.04 mm – 1.51 mm) for the right side. This is important as the inferior (i.e. midgland to apex) part of the NVB is in closest approximation to the prostate (Fig. 1). At that level the conflict between dose coverage of the prostate and dose sparing of the NVBs is highest. Due to the steep dose gradient, further away from the target volume the delivered dose will be progressively lower. Therefore, a lower agreement is acceptable for structures-to-spare at further distance from the prostate such as the IPA and the superior part of the NVB, opposed to structures closer to the prostate such as the inferior part of the NVB. Furthermore, we showed that agreement improved in the main study after the contouring atlas was updated and the MRI sequence was improved with knowledge gained from the pilot study, even though the 1.5 T modality was used for the main study opposed to the 3 T modality for the pilot study. We used the 1.5 T modality in the main study as it is the actual MRI used for daily treatment adaptation during MRgRT, which make the results better to translate into clinical practice. The substantial improvement in the main study suggests that further enhancement of MRI as well as ongoing training will lead to a better agreement in future assessment, which is needed for clinical implementation of neurovascular-sparing MRgRT.

In our experience the NVB is generally well identifiable at the prostate apex where it is delimited by the dorsolateral part of the prostate and the ventrolateral part of the rectum. Towards the base of the prostate, the NVB becomes more divergent and diffuse and therefore harder to distinguish (Fig. 2). Especially at the level of the vesicles identification and contouring is difficult and care should be taken that the NVBs are not confused with the seminal vesicles. In most cases the NVBs are located lateral to ventrolateral of the vesicles [2]. The IPAs were considered to be very well distinguishable on MRI throughout their entire trajectory. Starting from inferior up on the transverse plane, the artery makes a characteristic turn from lateral to ventral around the sacroiliac ligament entering the pelvis through the lesser sciatic foramen, then continuing and merging into the corpora cavernosa. Discordance of contours of the IPA in our study were mainly caused by the variance of width of the margin taken around the artery by the individual raters (Fig. 3) [2].

A limitation of this study is the limited non-random study sample. Anatomical variation of the NVB results in favorable and unfavorable variations for neurovascular-sparing radiotherapy [2]. The favorable variations are generally better to distinguish on MRI which could lead to a better agreement between raters. Although we contoured an unselected consecutive series of 15 patients in the main study, it remains unknown how these results compare to the general prostate cancer population anatomy and whether or not our series is relatively favorable or unfavorable, which might have caused an over- or underestimation of the interrater agreement. Furthermore, the contours of the NVB and IPA were adjusted to run from the same superior to inferior level. This was done because the NVB and IPA continue in superior direction far distant from the prostate and area of clinical relevance for neurovascular dose sparing. Large interrater variations of longitudinal contoured distance of the same structure can therefore skew results of measures of agreement. However, the adjusted superior to inferior distance varied between the contoured structures and patients, which explains the difference in volumes between the NVBs pilot and main study. Moreover, in the main study the contours were mainly extended towards the superior direction compared to the pilot study, where the NVB is more divergent and therefore generally contoured wider. The difference in contoured longitudinal distance of the NVB and IPA may induce a bias as contouring a longer distance results in more possibility of disagreement. Also, the superior part of the NVB is more difficult to contour, which became apparent from the results of our study. These factors may lead to a relatively lower DSC for greater longitudinal contoured distances for the NVB and IPA, which should be kept in mind when interpreting the results. Nevertheless, despite the generally greater longitudinal contoured distance in the main study compared to the pilot study, the agreement remained better in the main study.

## Conclusion

We found that the interrater agreement for the contouring of the NVB and IPA improved with enhancement of the MRI sequence as well as further training of the raters. The agreement was best in the subset of the inferior half of the NVB, where a good agreement is clinically most relevant for neurovascular-sparing MRgRT for PCa.

## Funding

This research has been partly funded by ZonMW IMDI/LSH-TKI Foundation (The Hague, The Netherlands, project number 104006004), Elekta AB (Stockholm, Sweden), and Philips Medical Systems (Best, The Netherlands). The funding sources had no involvement in the design of the study, the collection, analysis, and interpretation of the data, nor in the writing and decision to submit the article for publication.

## Declaration of Competing Interest

The authors declare the following financial interests/personal relationships which may be considered as potential competing interests: HV receives research funding from Elekta. The remaining authors declare no potential competing interests.
