# Supplementary material for: Interrater agreement of contouring of the neurovascular bundles and internal pudendal arteries in neurovascular-sparing magnetic resonance-guided radiotherapy for localized prostate cancer
Source: Clin Transl Radiat Oncol. 2021 Nov 14;32:29–34. doi: 10.1016/j.ctro.2021.11.005 (PMC8605225; doi:10.1016/j.ctro.2021.11.005)
Supplement: Supplementary data 1 [file mmc1.pdf]

UMC Utrecht

Contouring atlas

Version 1.0

Open studypatient in Volumetool Study

- Volumetool study -->
- study general -->
- patient -->
- open patient -->
- search: [patient number] -->
- Case: select MRL online

UMC Utrecht

Structures to contour

- Prostate (prostate) per protocol
- Neurovascular bundle left (NVB_le)
- Neurovascular bundle right (NVB_ri)
- Internal pudendal artery left (IPA_le)
- Internal pudendal artery right (IPA_ri)

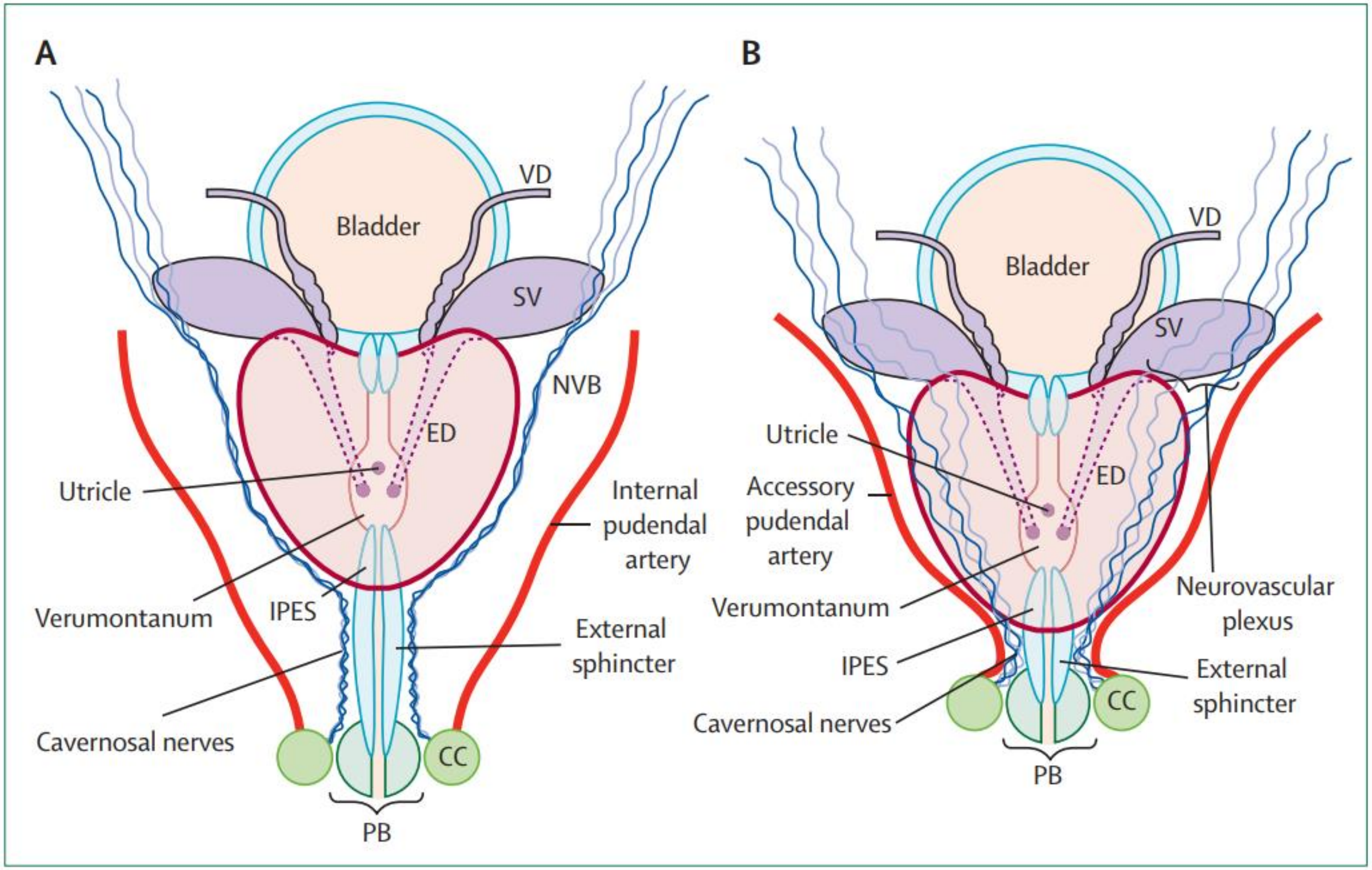

Figure 5: **Favourable and unfavourable variations in anatomy for preservation of erectile function after radiotherapy**

(A) The most favourable configuration with a classic well-defined neurovascular bundle, long external sphincter with adjacent cavernosal nerves, and a normal internal pudendal artery running far from the prostate and external sphincter. (B) Unfavourable variant anatomy includes a dispersed adherent neurovascular plexus, a short external sphincter, and the accessory pudendal artery variant running close to the prostate apex and external sphincter. CC=corpus cavernosa. ED=ejaculatory duct. IPES=intraprostate external sphincter. NVB=neurovascular bundle. PB=penile bulb. SV=seminal vesicles. VD=vas deferens.

UMC Utrecht

Source: Lee et al. 2016. Vessel-sparing radiation and functional anatomy-based preservation for erectile function after prostate radiotherapy

Neurovascular bundle

Standard abbreviation: NVB_le; NVB_ri

Nerves arise from the inferior hypogastric plexus from the lateral surface of the rectum and converge posterolateral to the prostate

- Before the nerves converge at the base, the plexus may have a spread of 3 cm. The most anterior nerves are close to the vesicles.
- The nerves diverge again at the posterolateral apex and pass through the urogenital diaphragm as several separate elements and then reach the corpera cavernosa

<u>Per protocol</u>

The NVB should be contoured from at least the base of the seminal vesicles until the level of the urogenital diaphragm.

UMC Utrecht

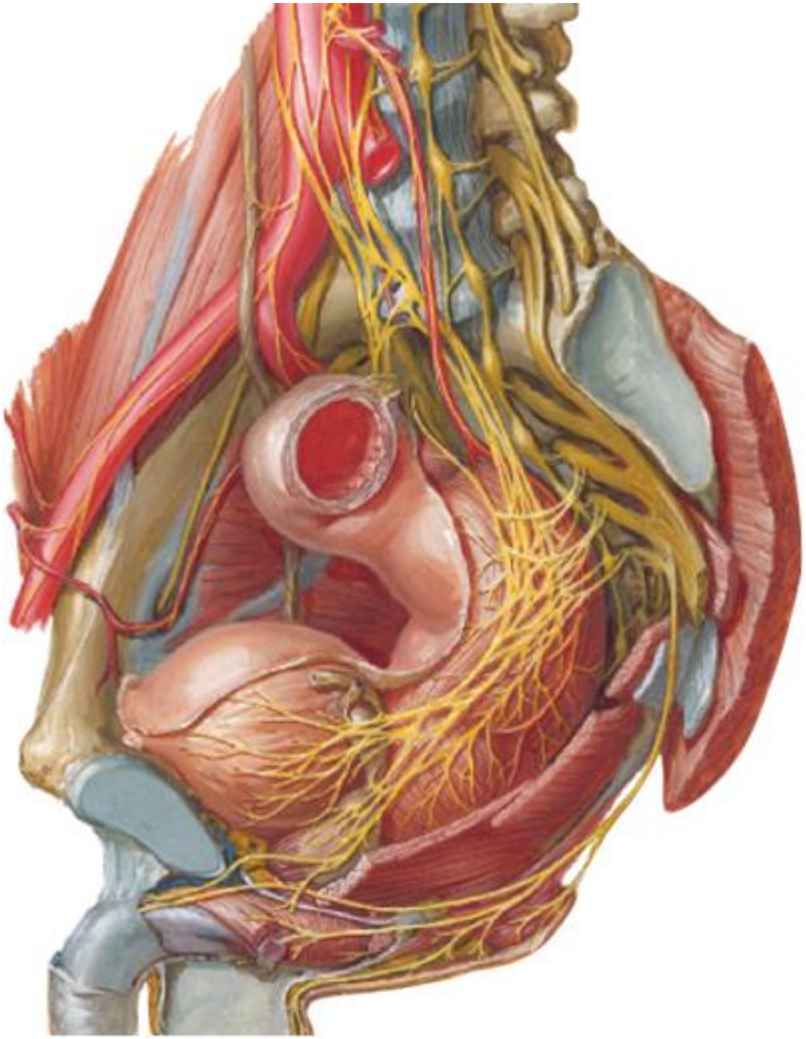

Source: Atlas of Human Anatomy, Netter, Frank H., M.D., 7th edition

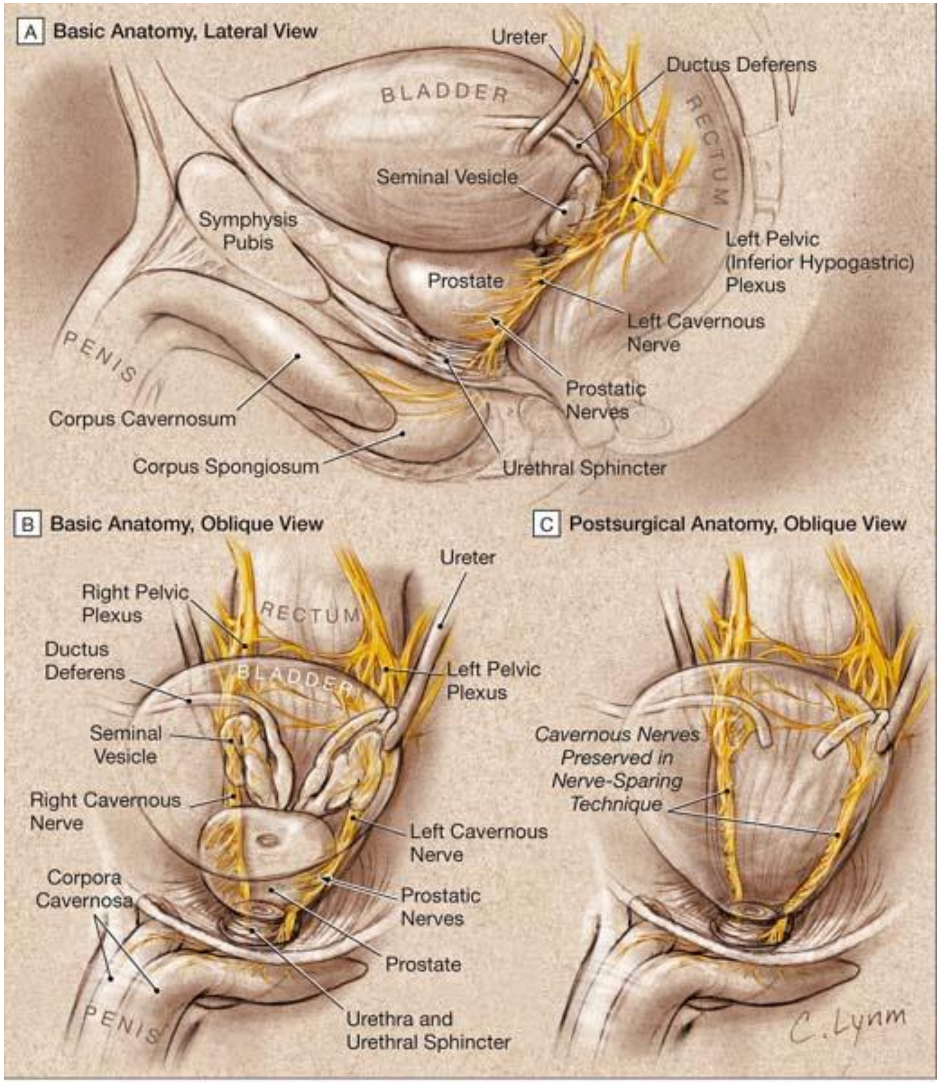

©2005 American Medical Association (all rights reserved) JAMA, June, 2005-Vol. 293, No 21, p 2650.

UMC Utrecht

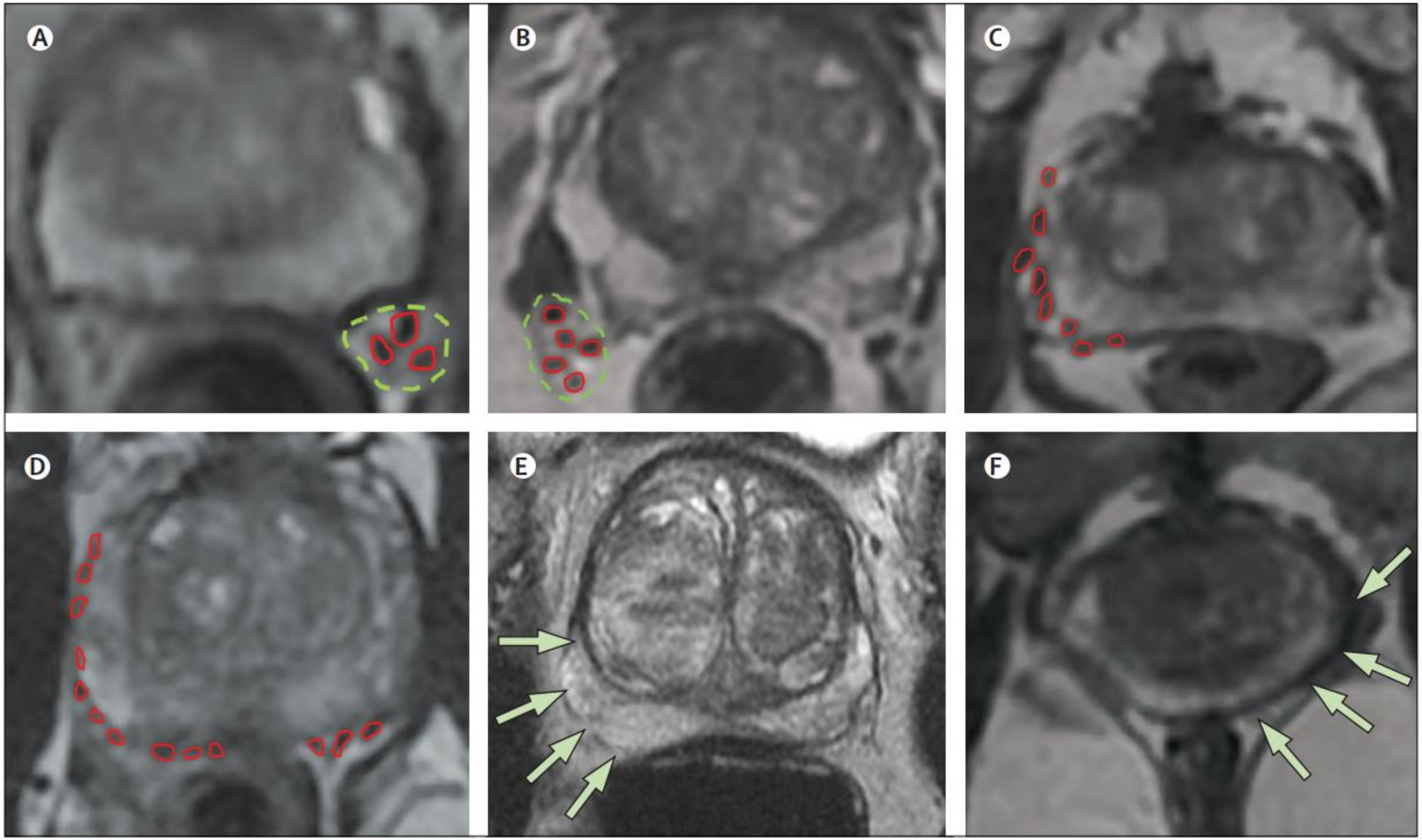

Figure 4**: Nerve bundle variation**

A and B show the classical neurovascular bundle near the posterolateral prostate. Green circles mark classic neurovascular bundles at the posterolateral prostate. Red circles mark individual neurovascular elements sometimes organised into bundles and sometimes distributed broadly around the prostate. C and D show the adherent nerve plexus pattern. E and F show the rare absent variant with no neurovascular elements.

UMC Utrecht

Source: Lee et al. 2016. Vessel-sparing radiation and functional anatomy-based preservation for erectile function after prostate radiotherapy

NVB contour example

The NVBs run posterolaterally of the seminal vesicles

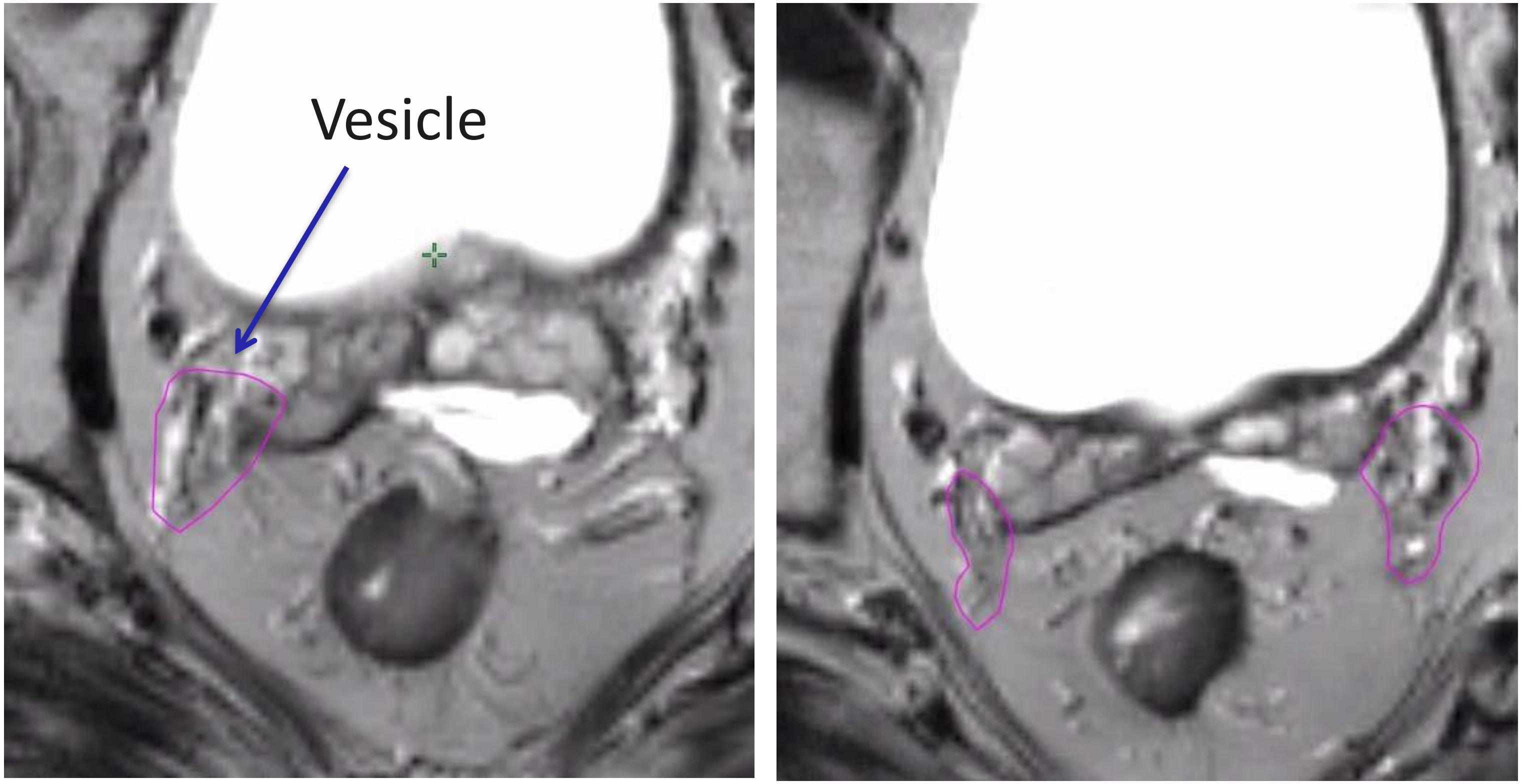

UMC Utrecht

Source: contouring atlas POTEN-C trial UT, Leiker, A & Desai, NB UTSW, 6/26/2018

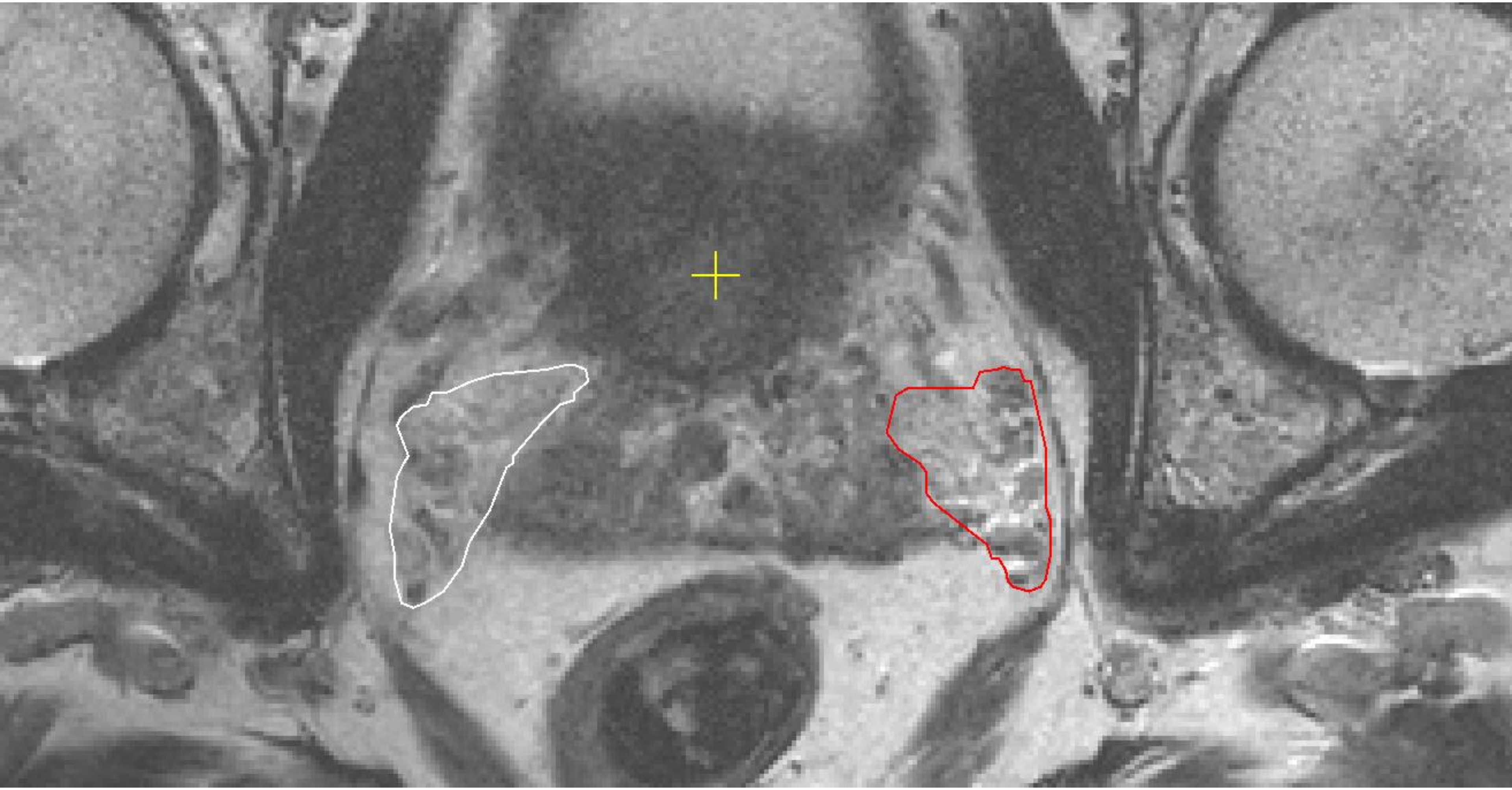

3T T2 MRI UMCU

Towards inferior the NVBs run dorsally against the peripheral zone of the prostate

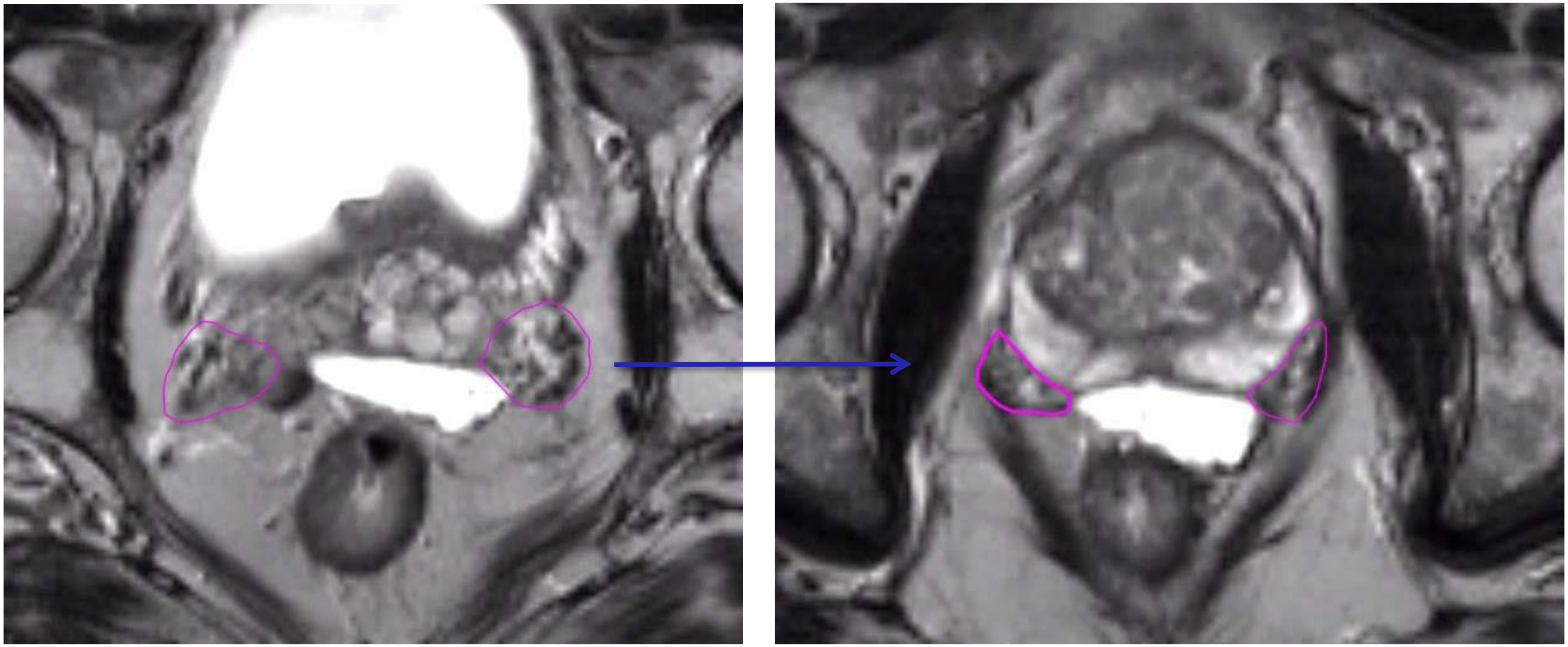

UMC Utrecht

source: contouring atlas POTEN-C trial UT, Leiker, A & Desai, NB UTSW, 6/26/2018

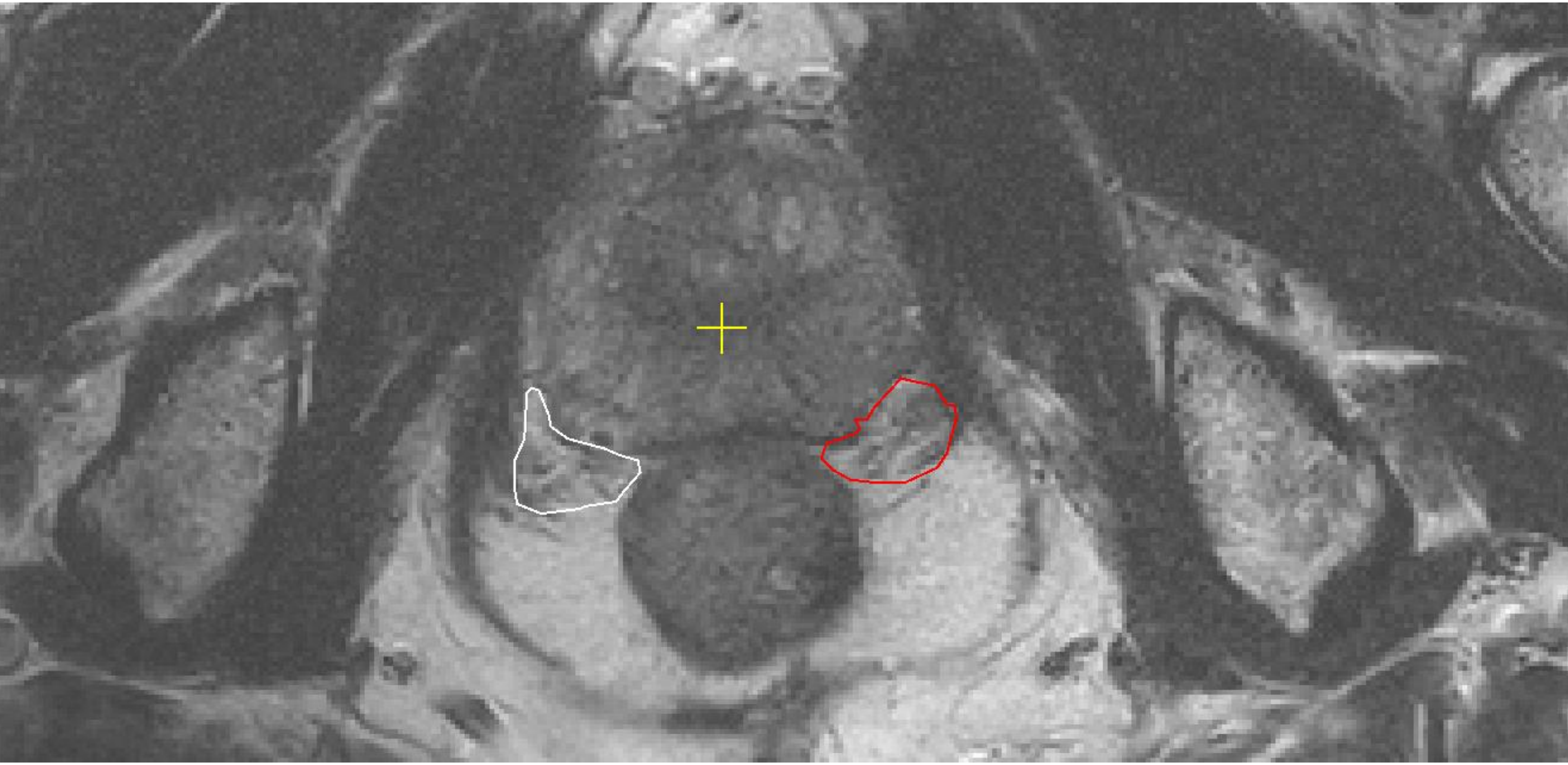

3T T2 MRI UMCU

Apex prostate

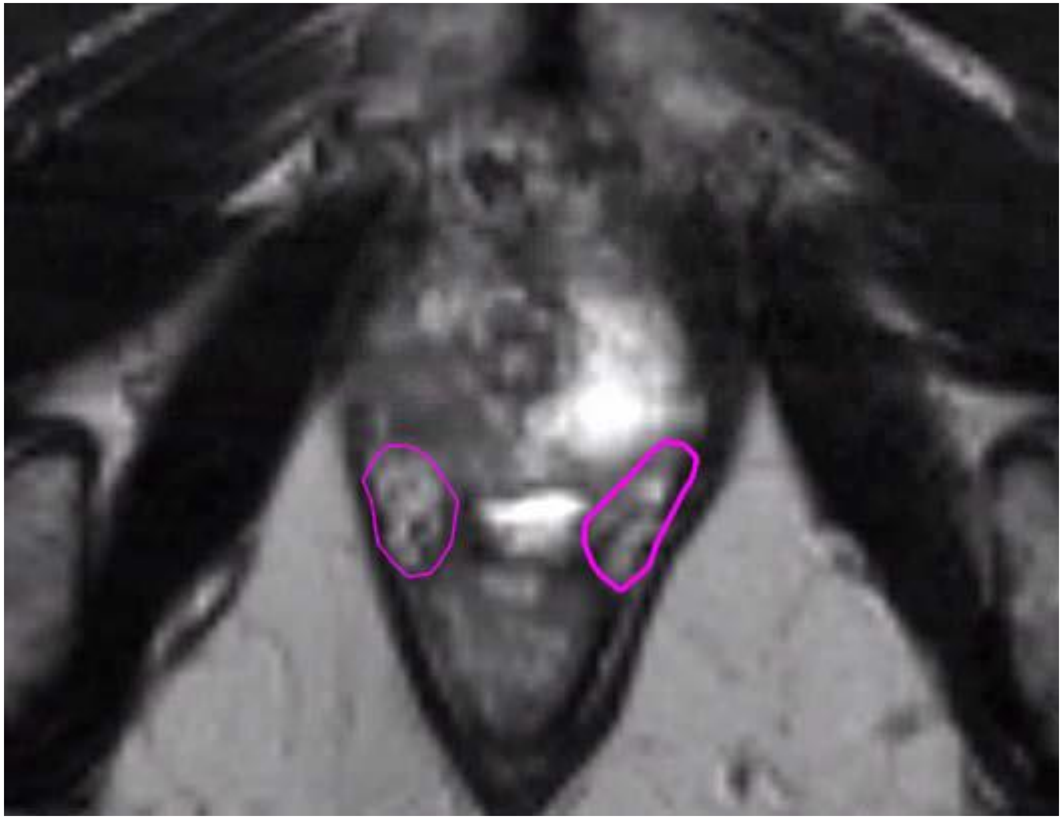

Source: *contouring atlas POTEN-C trial UT, Leiker, A & Desai, NB UTSW, 6/26/2018*

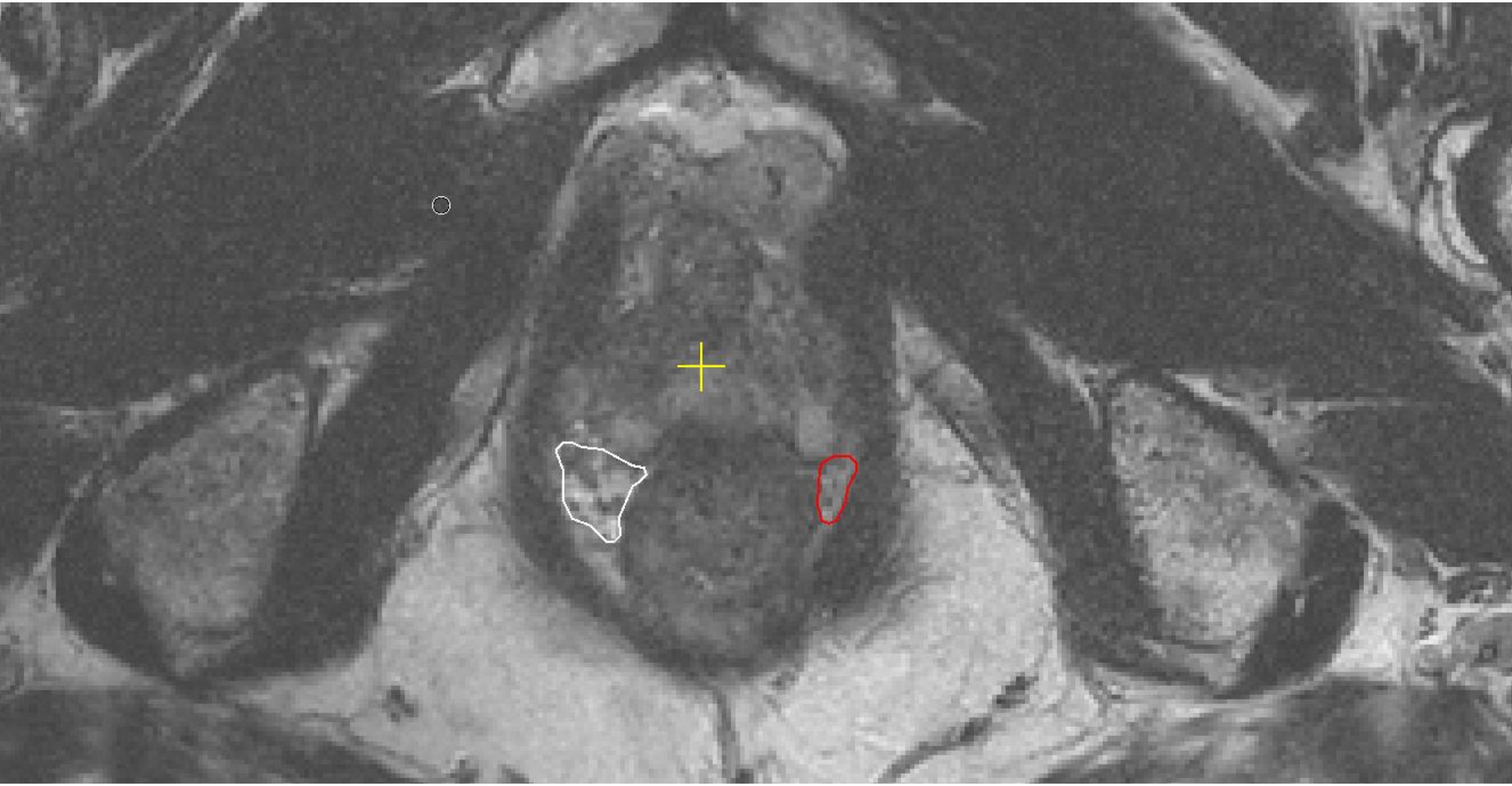

3T T2 MRI UMCU

The nerves diverge again at the posterolateral apex and pass through the urogenital diaphragm as several separate elements

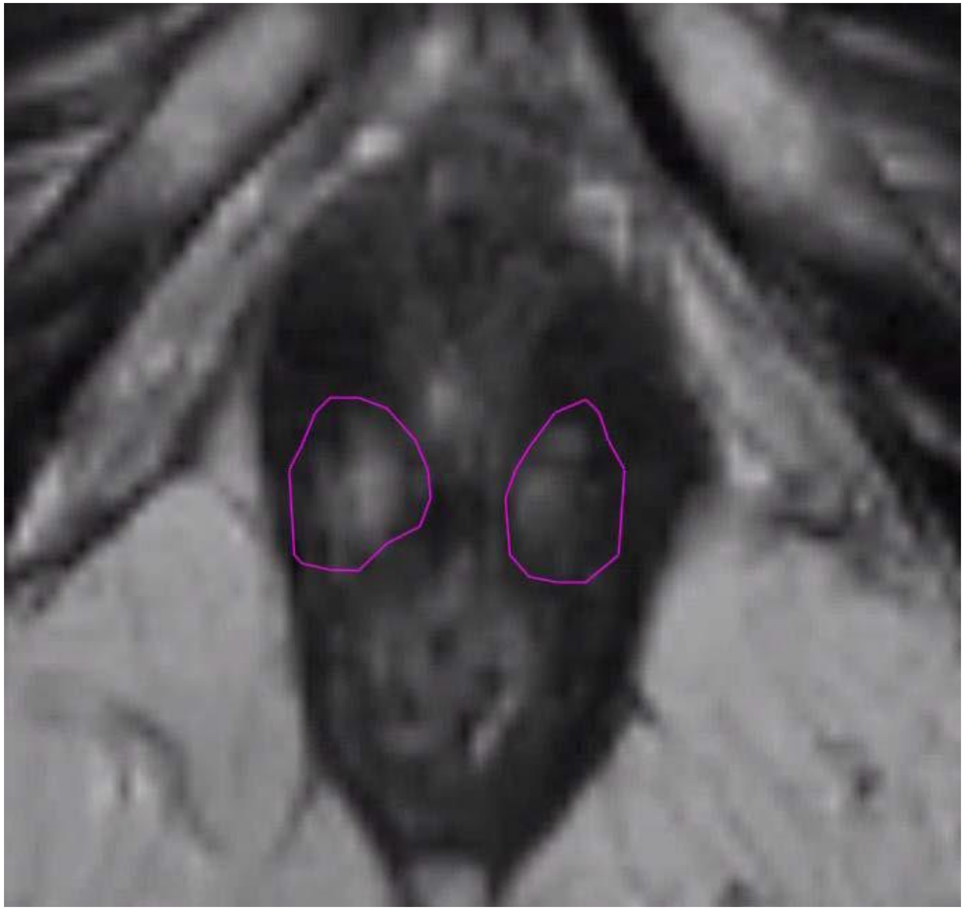

Source: contouring atlas POTEN-C trial UT, Leiker, A & Desai, NB UTSW, 6/26/2018

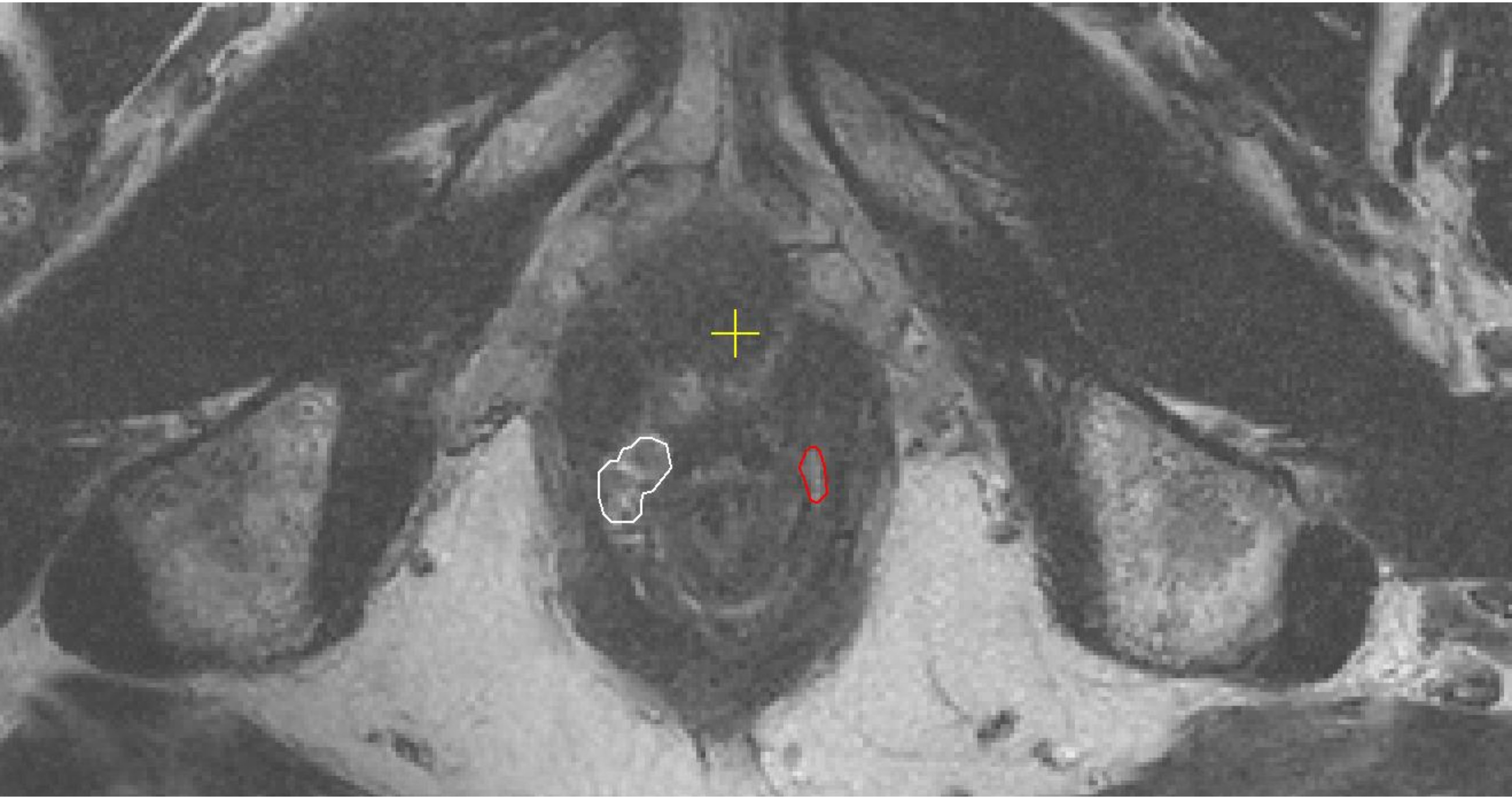

3T T2 MRI UMCU

Coronal plane: NVB right

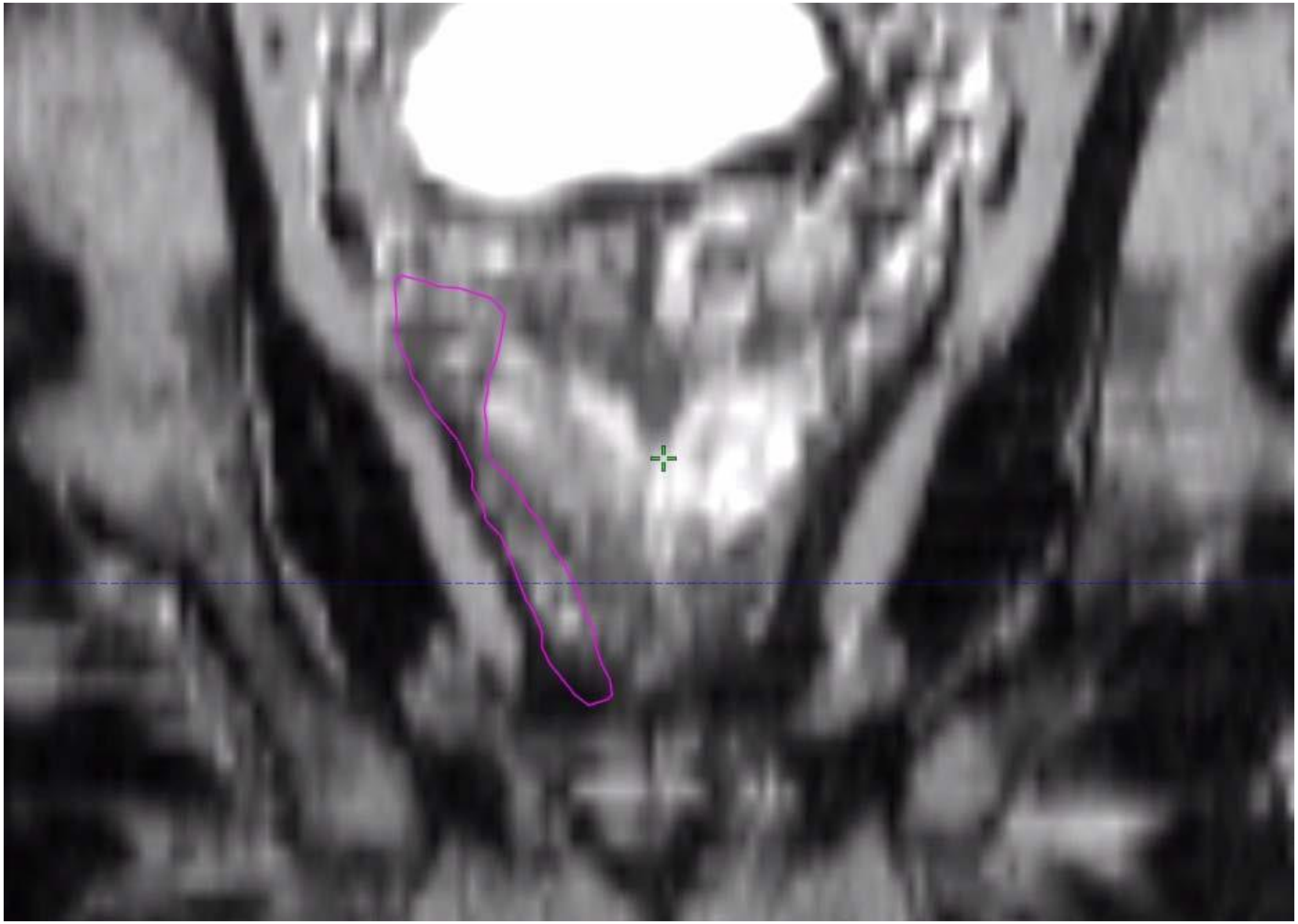

UMC Utrecht

Source: contouring atlas POTEN-C trial UT, Leiker, A & Desai, NB UTSW, 6/26/2018

Internal pudendal artery

Standard abbreviation: IPA_le; IPA_ri

The IPA branches from the internal iliac artery posterolaterally in the pelvis and exits the pelvis caudally to the superior gluteal artery at the inferior aspect of the greater sciatic foramen. The IPA takes a sharp turn under the sacroiliac ligament and is there easy to identify.

<u>Per protocol</u>

- The IPA should be contoured from at least the level of the sacroiliac ligament until the crus where it terminates into the common penile artery and the scrotal artery.

Internal pudendal artery

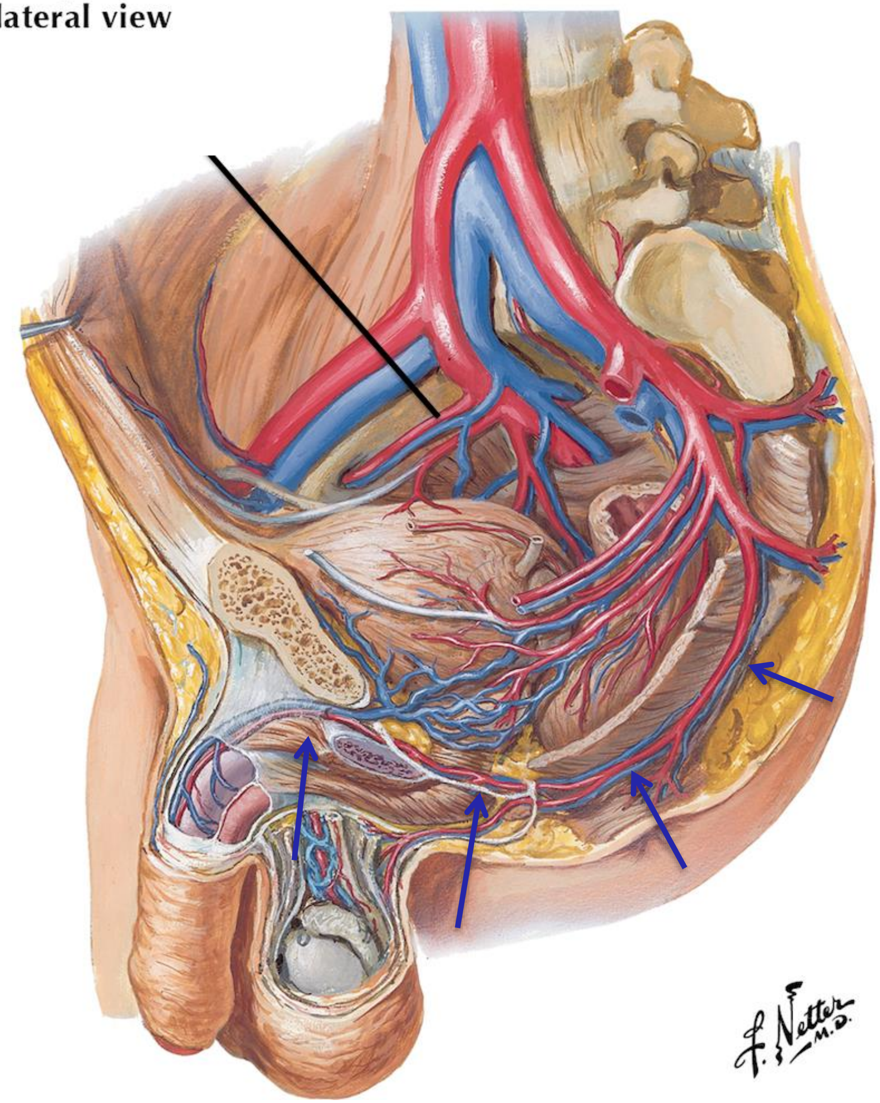

Source: Atlas of Human Anatomy, Netter, Frank H., M.D., 7th edition

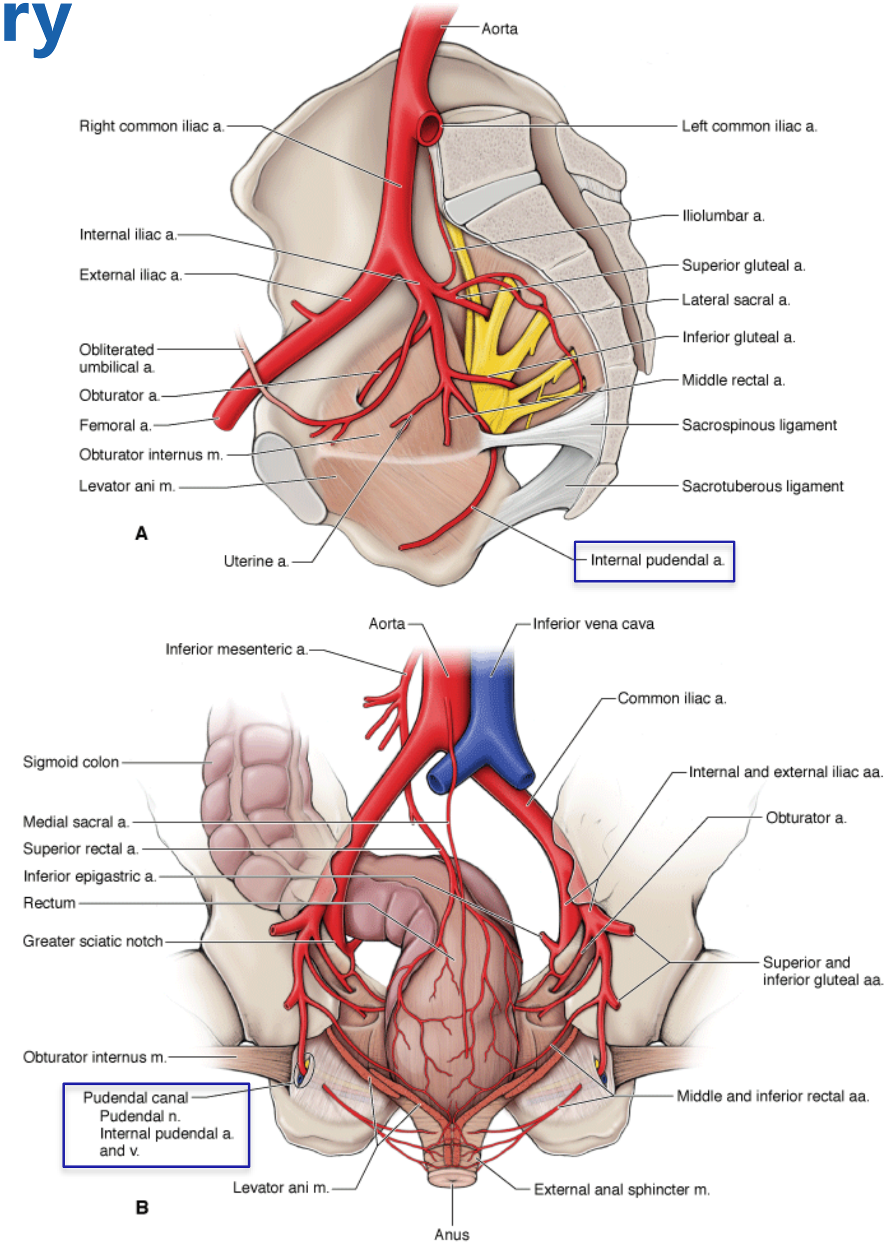

Source: Morton DA, Foreman KB, Albertine KH: *The Big Picture: Gross Anatomy:* www.accessmedicine.com

Copyright © The McGraw-Hill Companies, Inc. All rights reserved.

IPA descends

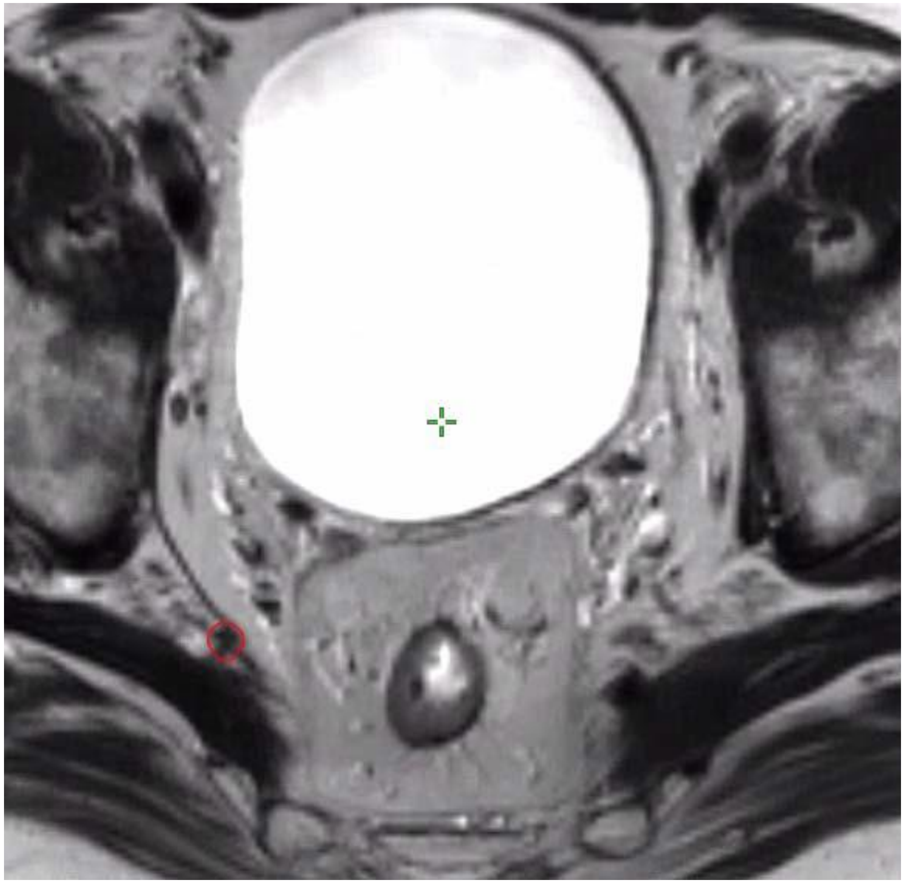

exits the greater sciatic foramen

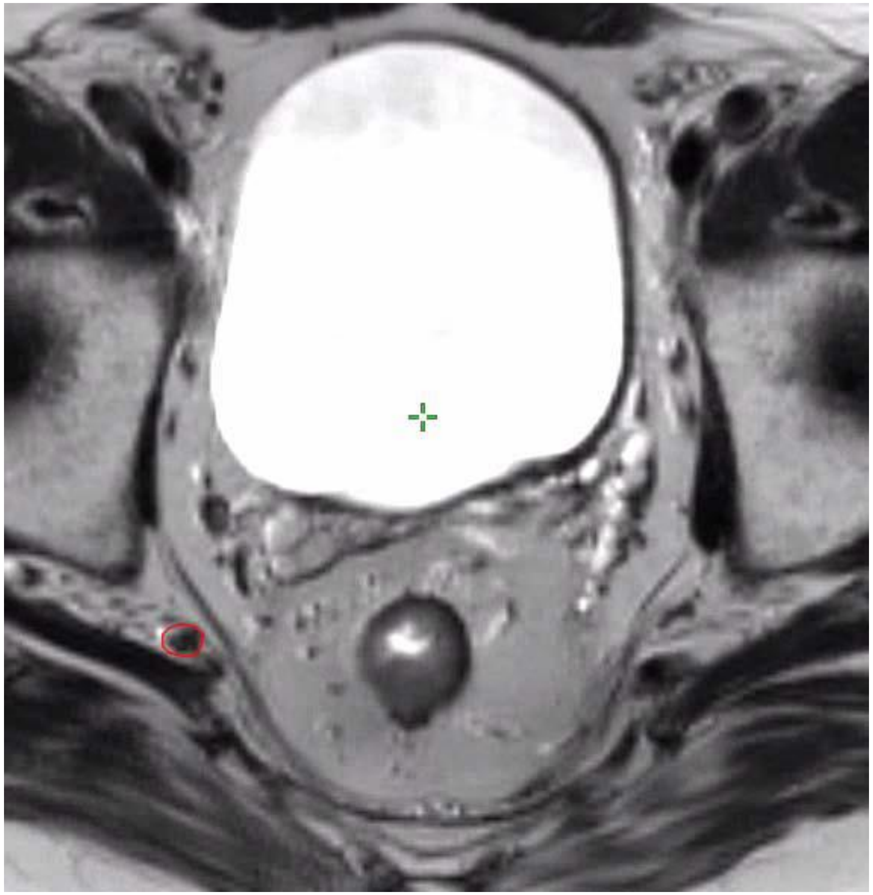

Towards inferior

UMC Utrecht

Source : contouring atlas POTEN-C trial UT, Leiker, A & Desai, NB UTSW, 6/26/2018

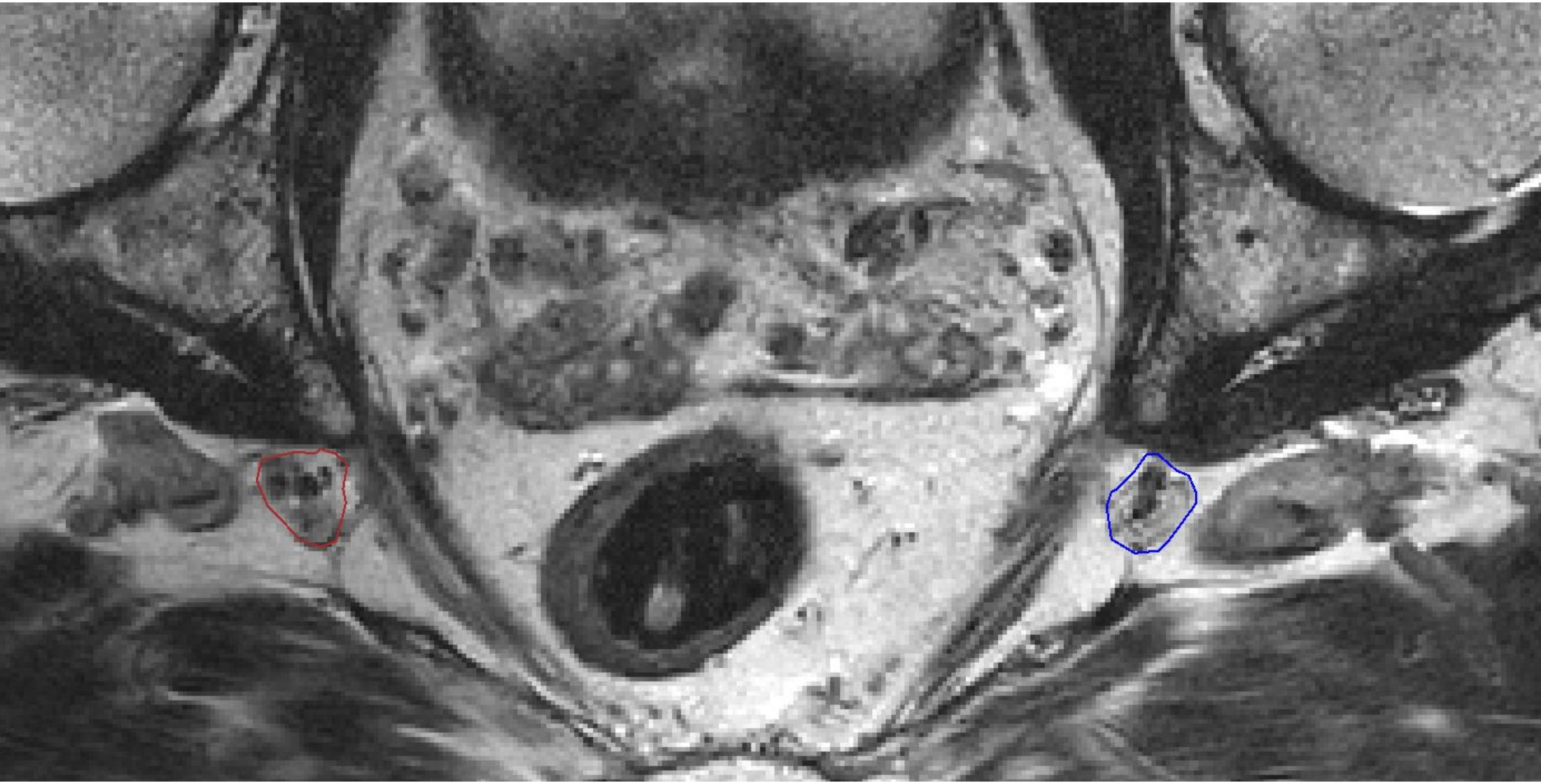

3T T2 MRI UMCU

IPA reaches the pelvis via the lesser sciatic foramen

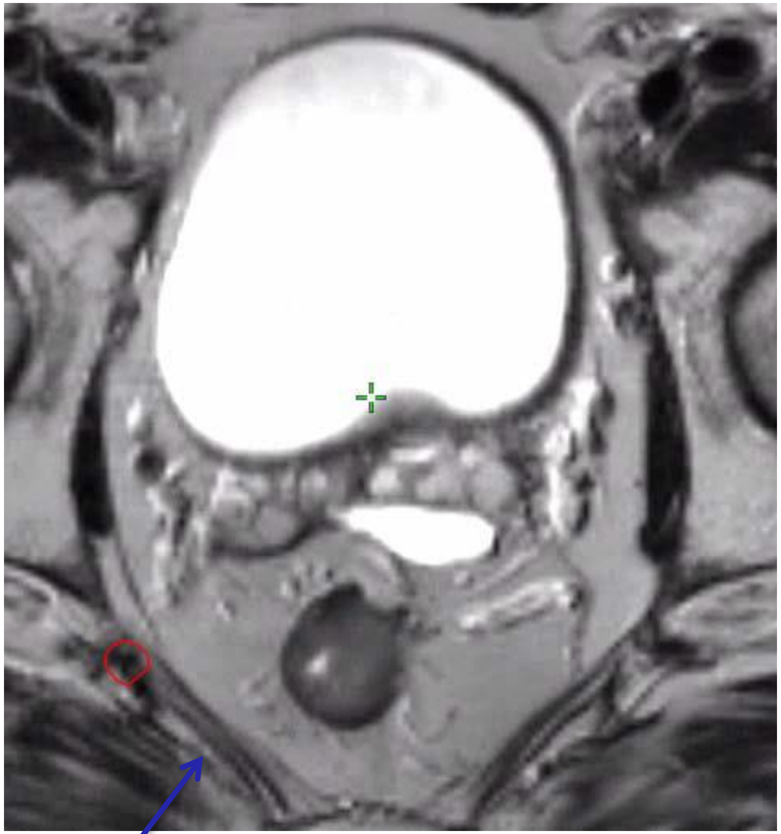

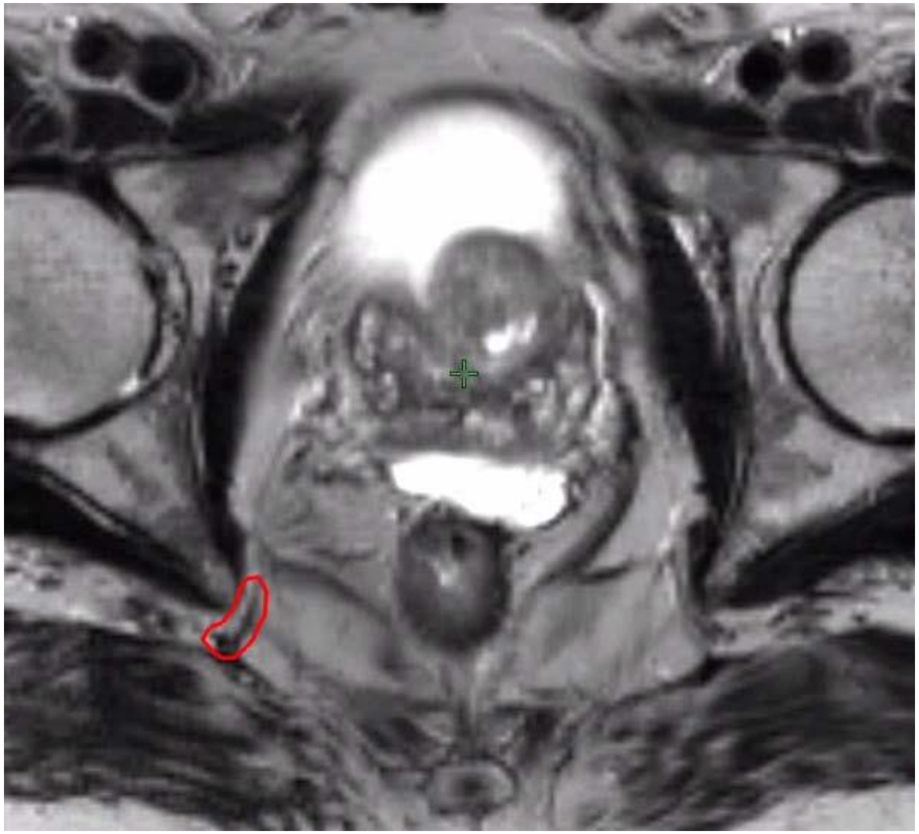

Sacroiliac ligament

IPA takes a sharp turn under the sacroiliac ligament

UMC Utrecht

Source : contouring atlas POTEN-C trial UT, Leiker, A & Desai, NB UTSW, 6/26/2018

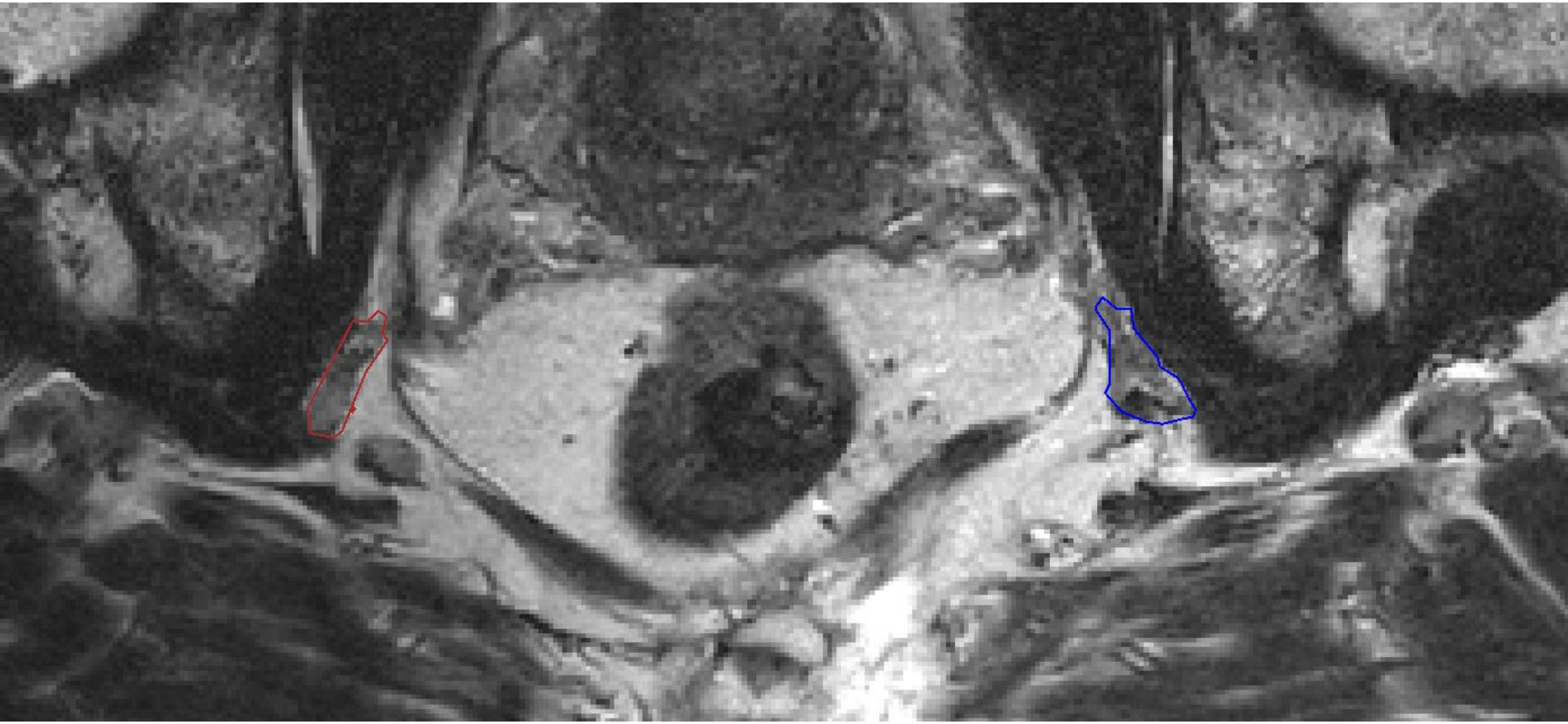

UMC Utrecht

3T T2 MRI UMCU

IPA ends at the level of the crus

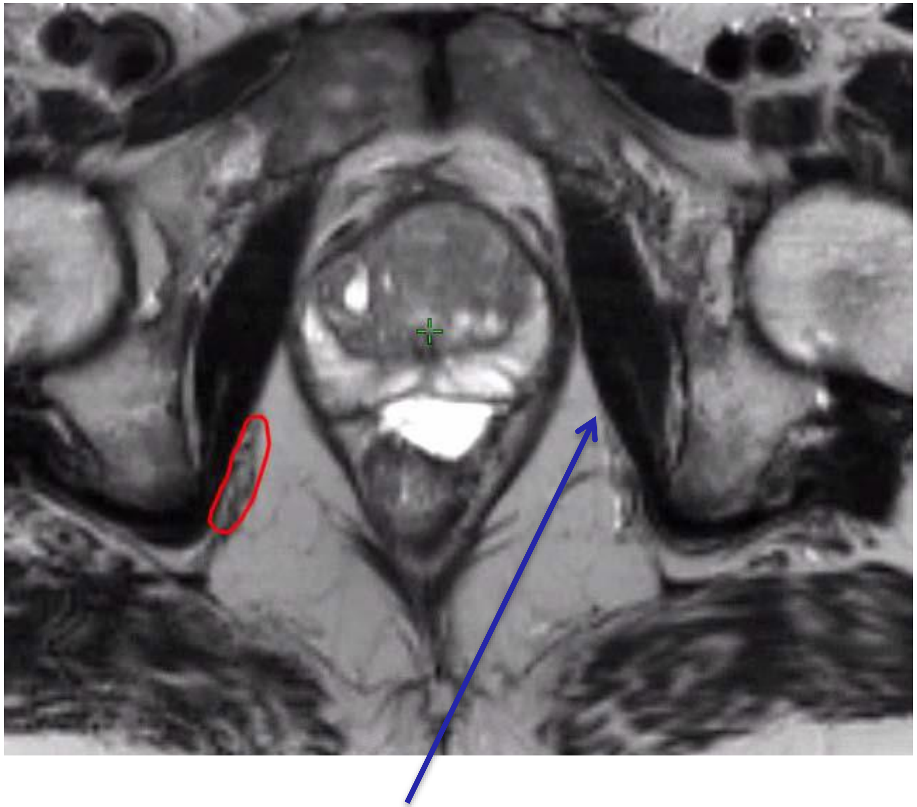

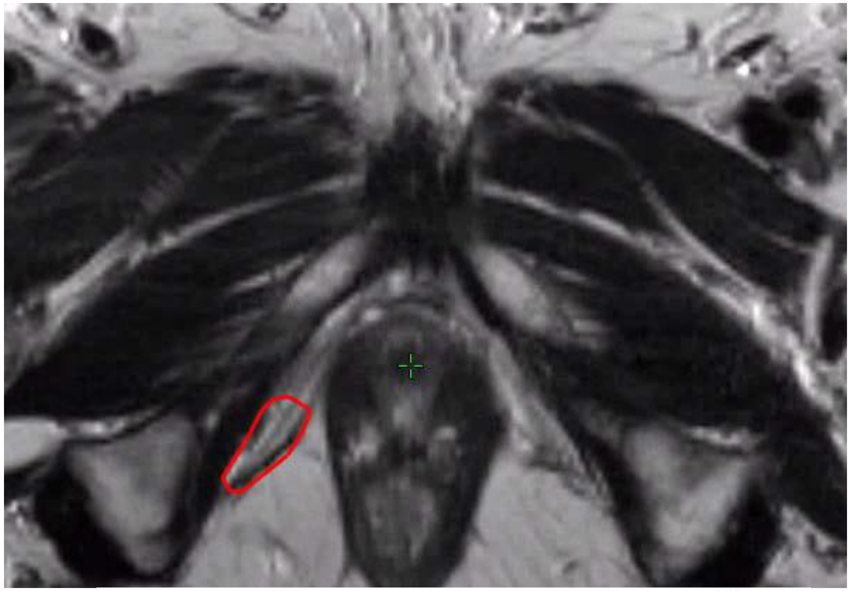

Internal obturator muscle

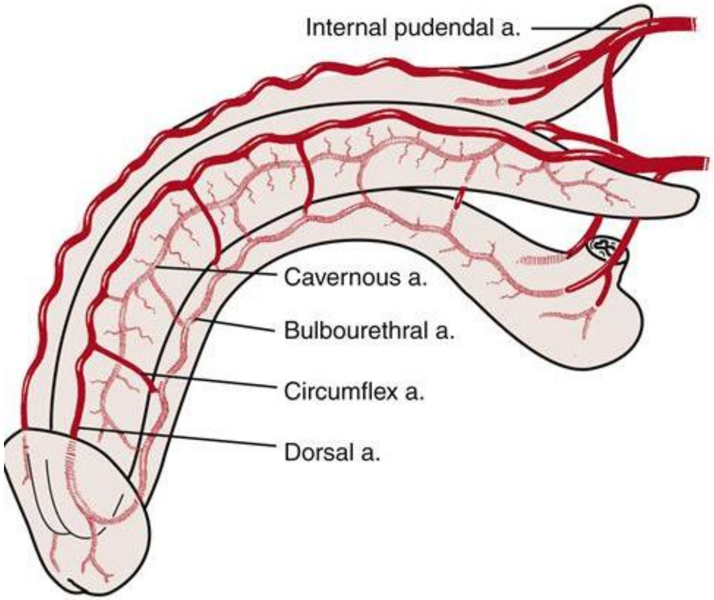

cht

Source : contouring atlas POTEN-C trial UT, Leiker, A & Desai, NB UTSW, 6/26/2018

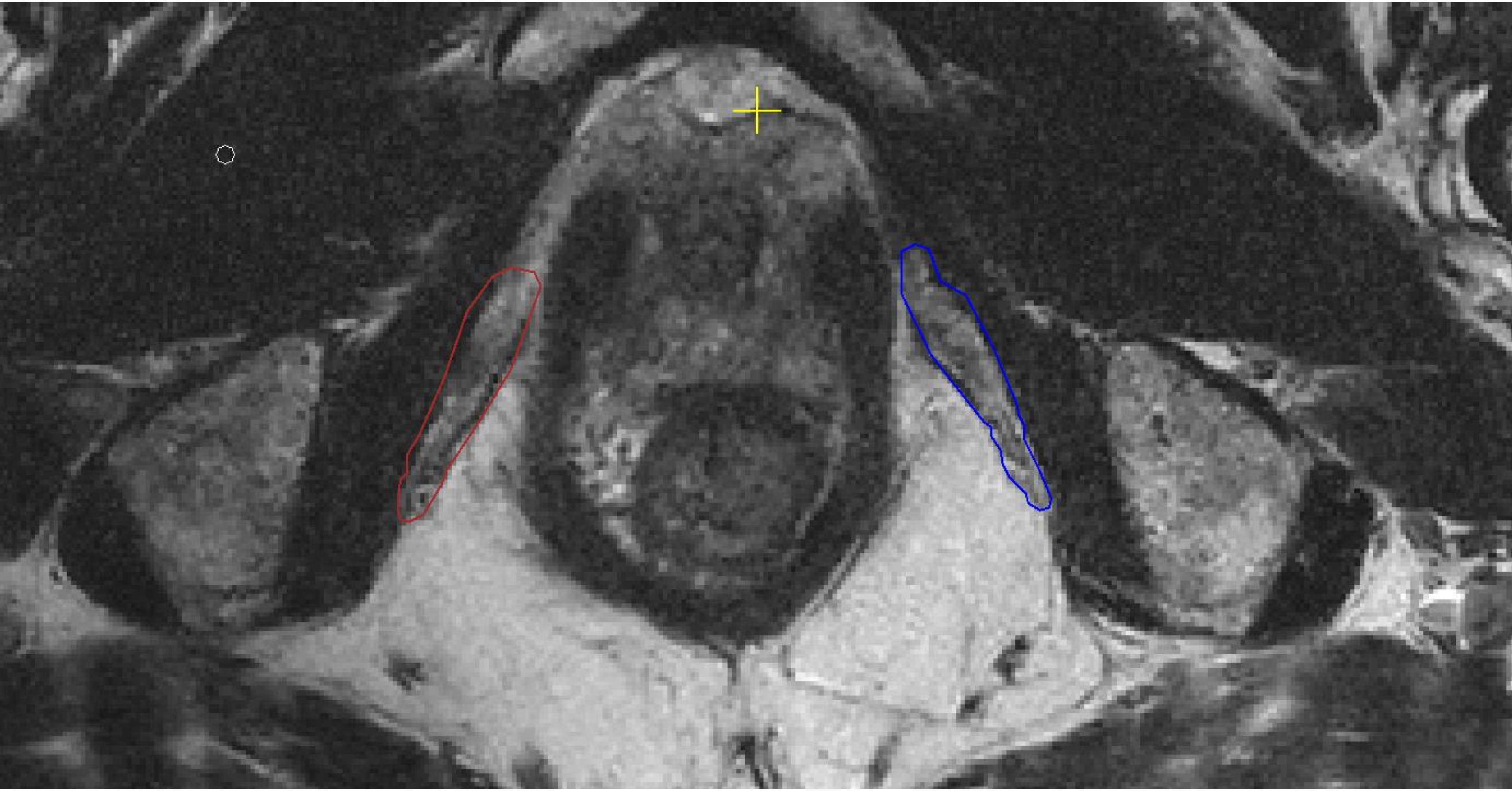

UMC Utrecht

3T T2 MRI UMCU

N.B.

- Contour NVB and IPA both left and right
- Make sure the NVB and prostate contours do not ovelap

UMC Utrecht
