# Supplementary material for: Interrater agreement of contouring of the neurovascular bundles and internal pudendal arteries in neurovascular-sparing magnetic resonance-guided radiotherapy for localized prostate cancer
Source: Clin Transl Radiat Oncol. 2021 Nov 14;32:29–34. doi: 10.1016/j.ctro.2021.11.005 (PMC8605225; doi:10.1016/j.ctro.2021.11.005)

Evaluation of pilot study

- To be used in addition to the contouring atlas version 1.0

Internal pudendal artery (IPA)

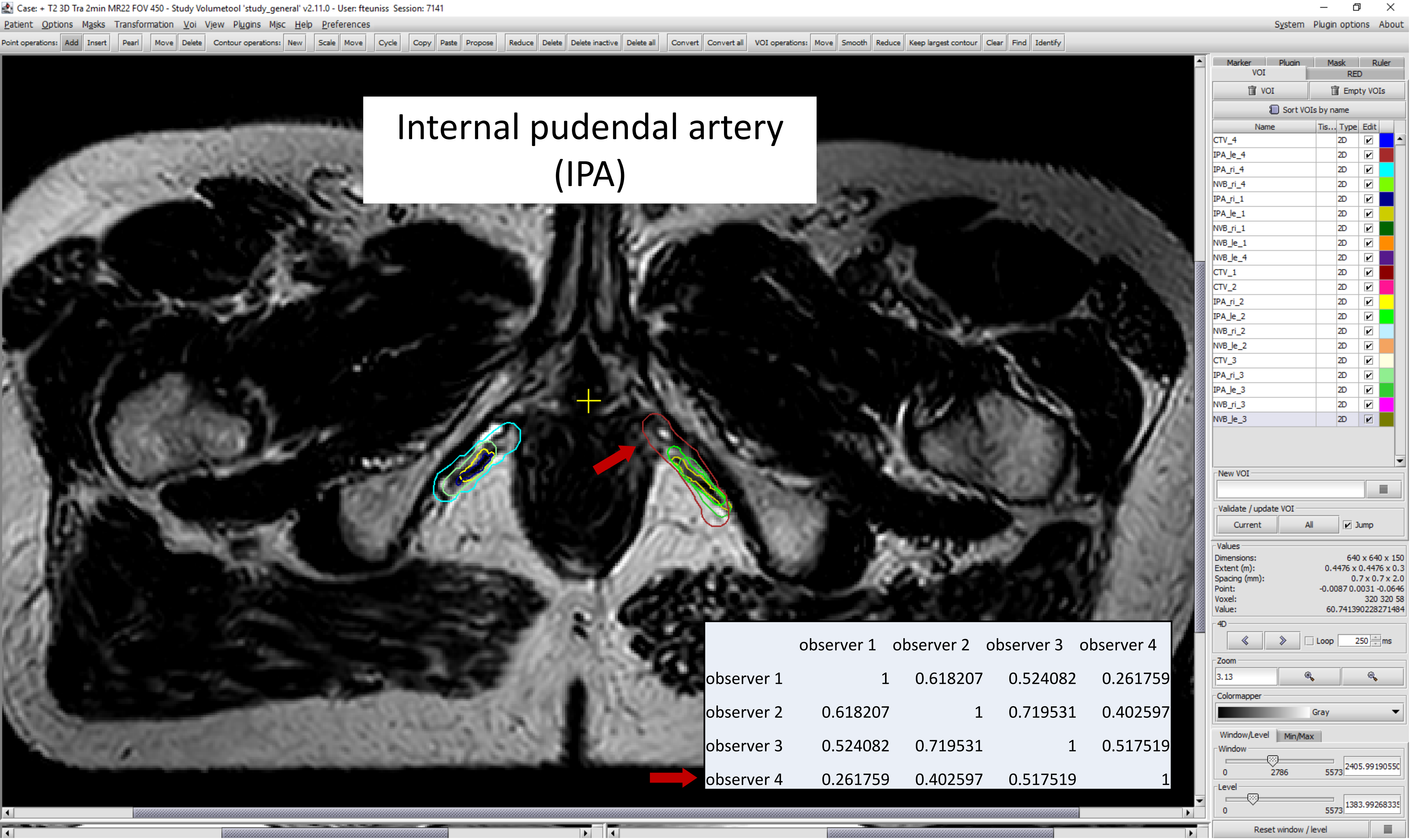

	observer 1	observer 2	observer 3	observer 4
observer 1	1	0.618207	0.524082	0.261759
observer 2	0.618207	1	0.719531	0.402597
observer 3	0.524082	0.719531	1	0.517519
observer 4	0.261759	0.402597	0.517519	1

Internal pudendal artery (IPA)

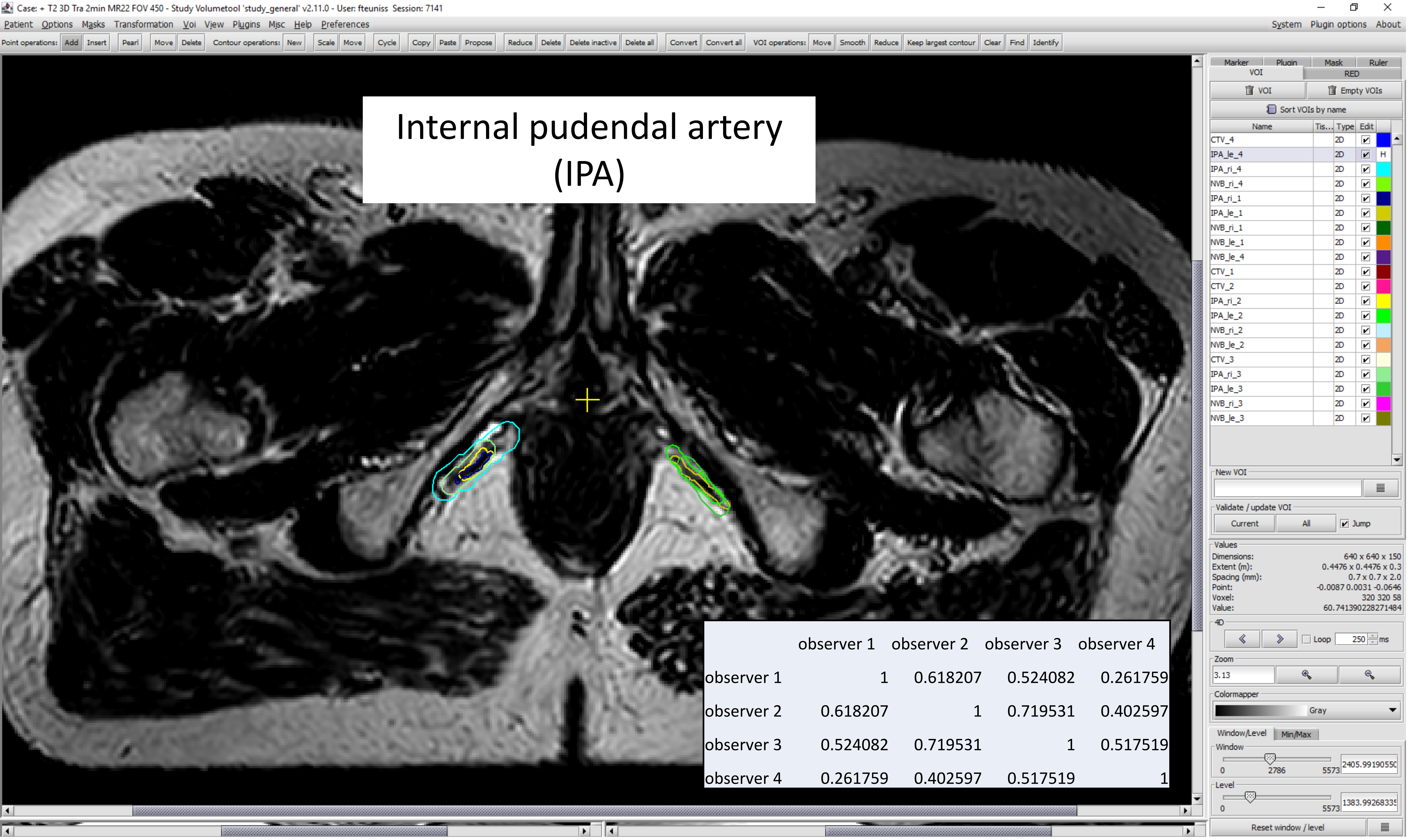

	observer 1	observer 2	observer 3	observer 4
observer 1	1	0.618207	0.524082	0.261759
observer 2	0.618207	1	0.719531	0.402597
observer 3	0.524082	0.719531	1	0.517519
observer 4	0.261759	0.402597	0.517519	1

Internal pudendal artery (IPA)

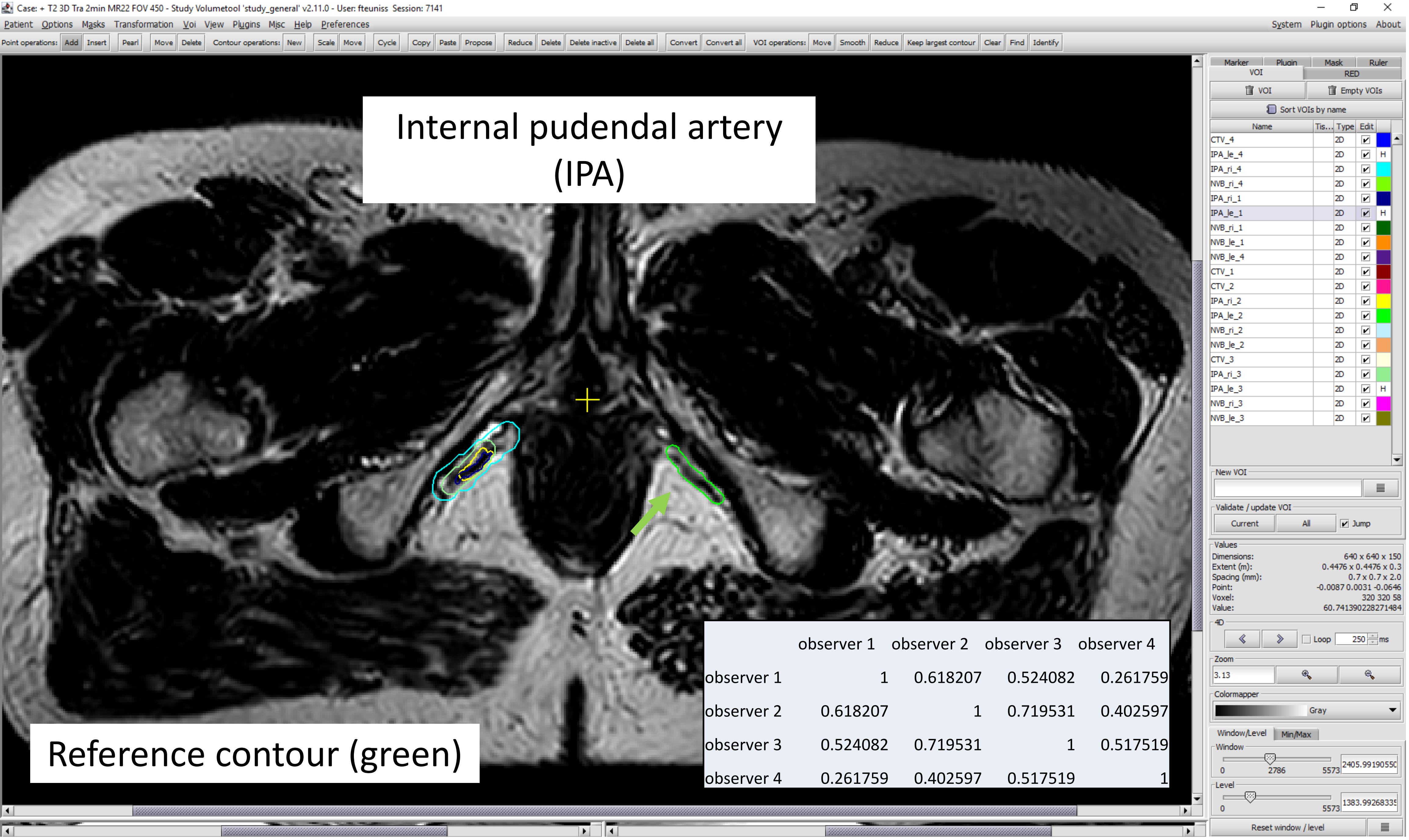

Reference contour (green)

	observer 1	observer 2	observer 3	observer 4
observer 1	1	0.618207	0.524082	0.261759
observer 2	0.618207	1	0.719531	0.402597
observer 3	0.524082	0.719531	1	0.517519
observer 4	0.261759	0.402597	0.517519	1

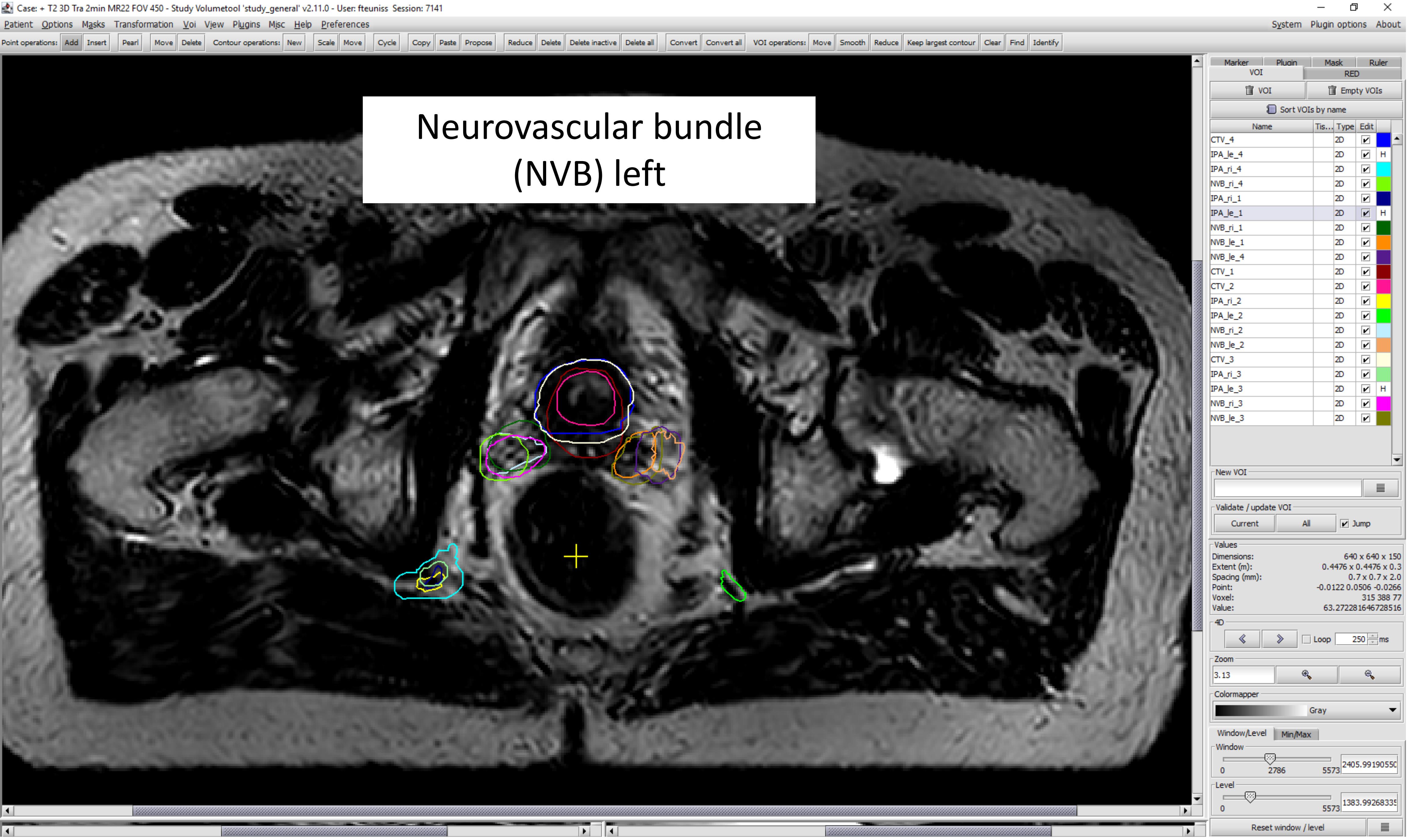
Case: + T2 3D Tra 2min MR22 FOV 450 - Study Volumetool 'study_general' v2.11.0 - User: fteuniss Session: 7141
Patient Options Masks Transformation Voi View Plugins Misc Help Preferences
System Plugin options About
Point operations: Add Insert Pearl Move Delete Contour operations: New Scale Move Cycle Copy Paste Propose Reduce Delete Delete inactive Delete all Convert Convert all VOI operations: Move Smooth Reduce Keep largest contour Clear Find Identify
Neurovascular bundle
(NVB) left
Marker Plugin Mask Ruler
VOI RED
VOI Empty VOIs
Sort VOIs by name
Name Tis... Type Edit
CTV_4 2D
IPA_le_4 2D H
IPA_ri_4 2D
NVB_ri_4 2D
IPA_ri_1 2D
IPA_le_1 2D H
NVB_ri_1 2D
NVB_le_1 2D
NVB_le_4 2D
CTV_1 2D
CTV_2 2D
IPA_ri_2 2D
IPA_le_2 2D
NVB_ri_2 2D
NVB_le_2 2D
CTV_3 2D
IPA_ri_3 2D
IPA_le_3 2D H
NVB_ri_3 2D
NVB_le_3 2D
New VOI
Validate / update VOI
Current All Jump
Values
Dimensions: 640 x 640 x 150
Extent (m): 0.4476 x 0.4476 x 0.3
Spacing (mm): 0.7 x 0.7 x 2.0
Point: -0.0122 0.0506 -0.0266
Voxel: 315 388 77
Value: 63.272281646728516
4D
Loop 250 ms
Zoom
3.13
Colormapper
Gray
Window/Level Min/Max
Window
0 2786 5573 2405.99190550
Level
0 5573 1383.99268335
Reset window / level

Neurovascular bundle (NVB) left

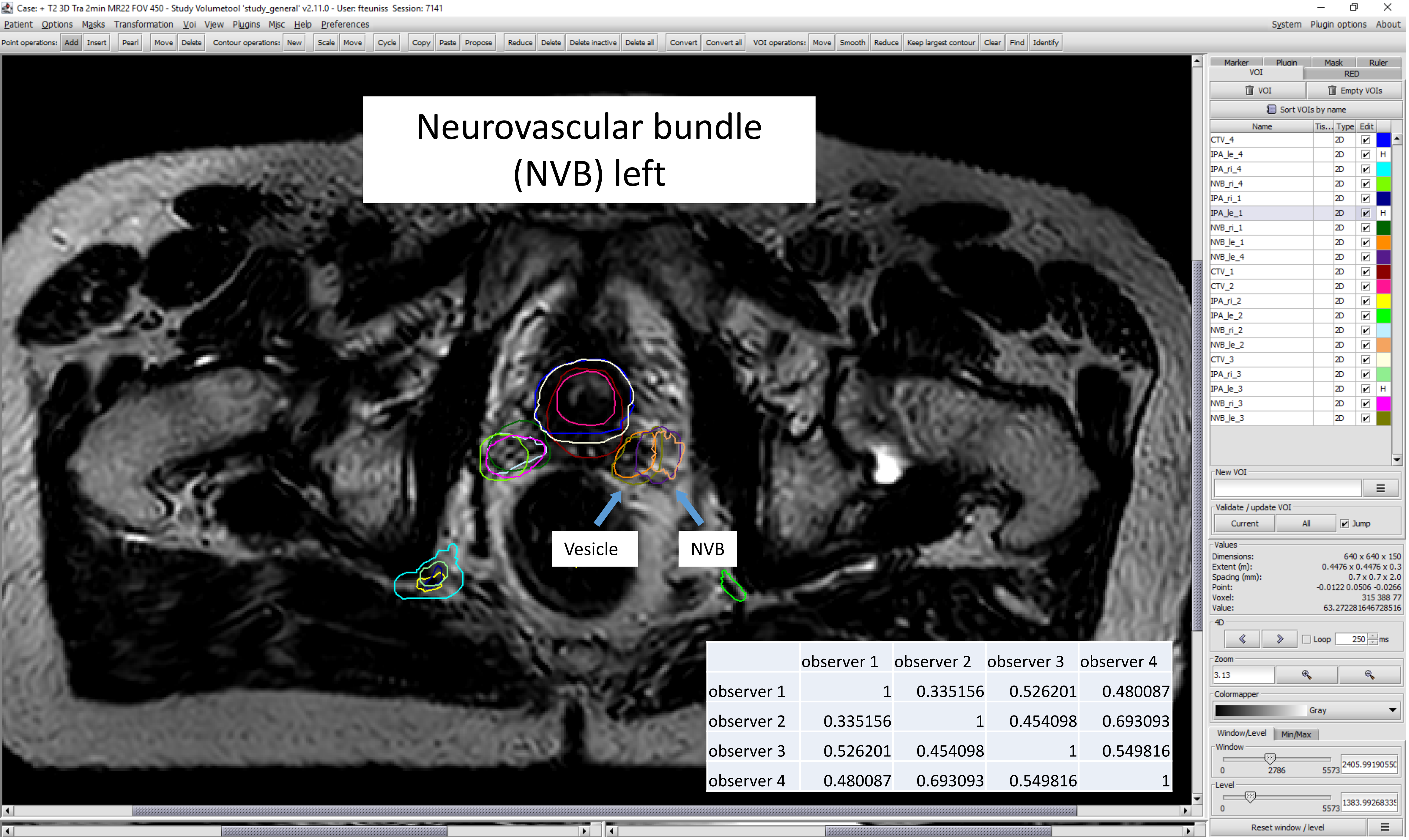

	observer 1	observer 2	observer 3	observer 4
observer 1	1	0.335156	0.526201	0.480087
observer 2	0.335156	1	0.454098	0.693093
observer 3	0.526201	0.454098	1	0.549816
observer 4	0.480087	0.693093	0.549816	1

Neurovascular bundle (NVB) left

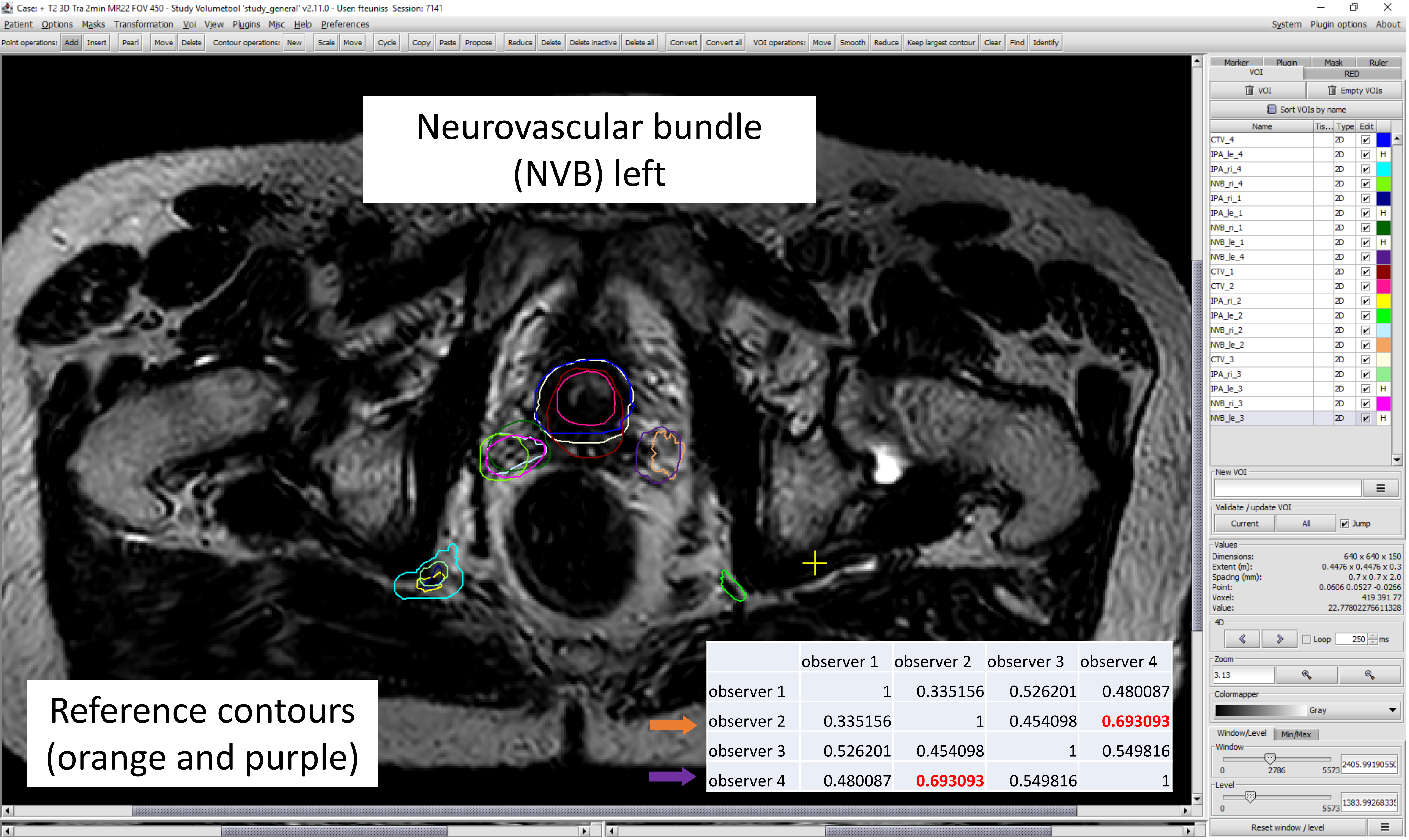

	observer 1	observer 2	observer 3	observer 4
observer 1	1	0.335156	0.526201	0.480087
observer 2	0.335156	1	0.454098	**0.693093**
observer 3	0.526201	0.454098	1	0.549816
observer 4	0.480087	**0.693093**	0.549816	1

Reference contours (orange and purple)

Neurovascular bundle (NVB) left

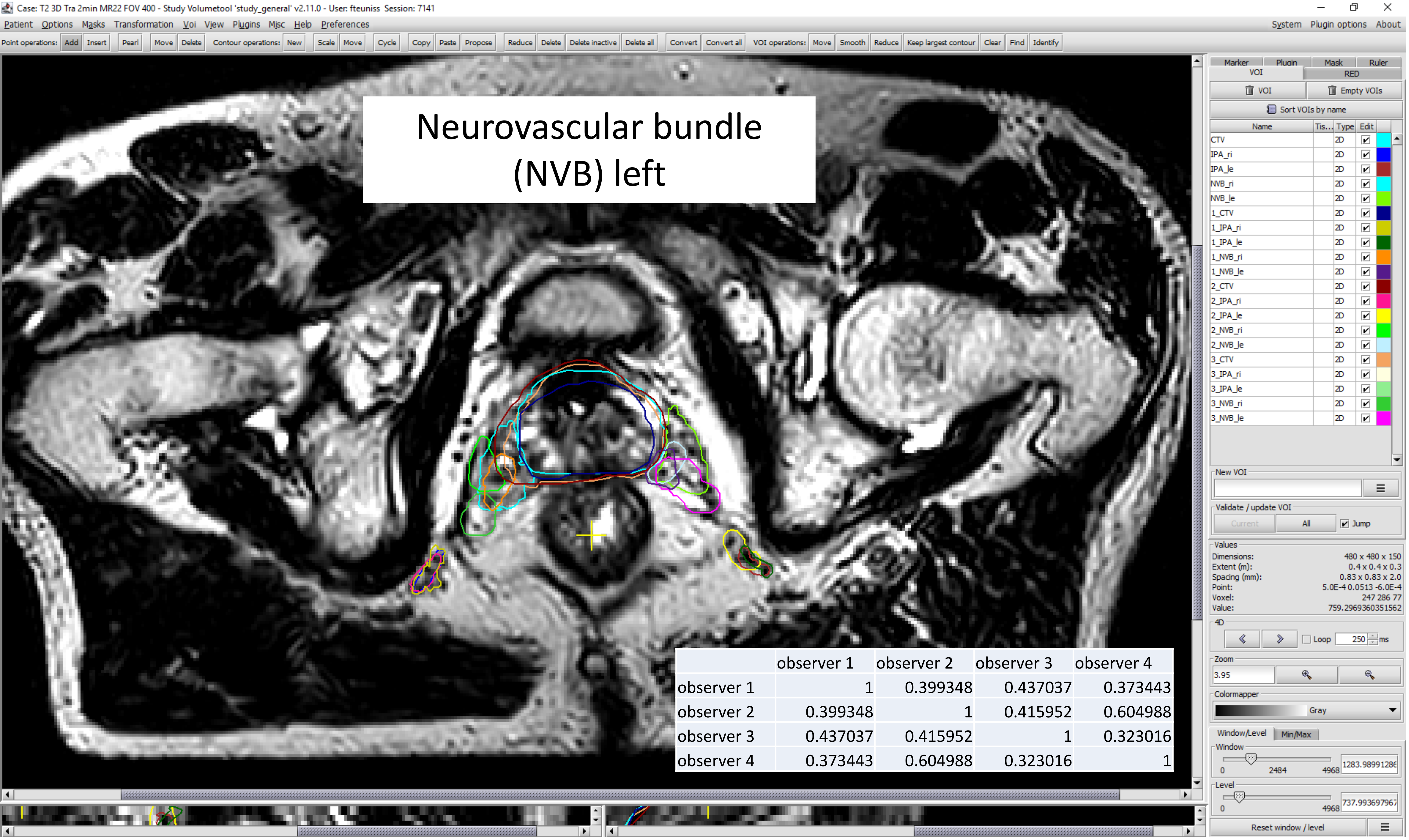

	observer 1	observer 2	observer 3	observer 4
observer 1	1	0.399348	0.437037	0.373443
observer 2	0.399348	1	0.415952	0.604988
observer 3	0.437037	0.415952	1	0.323016
observer 4	0.373443	0.604988	0.323016	1

Neurovascular bundle (NVB) left

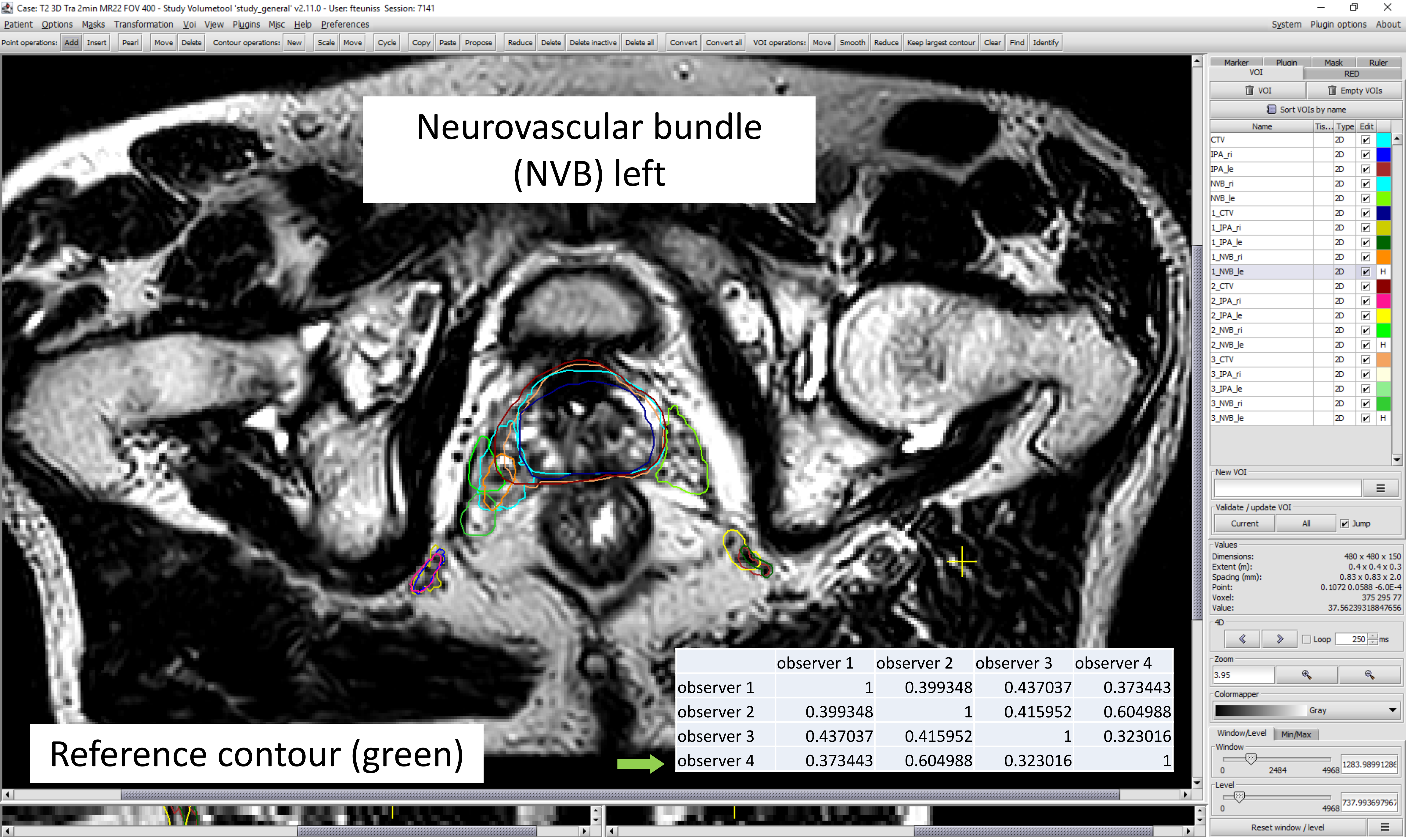

	observer 1	observer 2	observer 3	observer 4
observer 1	1	0.399348	0.437037	0.373443
observer 2	0.399348	1	0.415952	0.604988
observer 3	0.437037	0.415952	1	0.323016
observer 4	0.373443	0.604988	0.323016	1

Neurovascular bundle (NVB) left

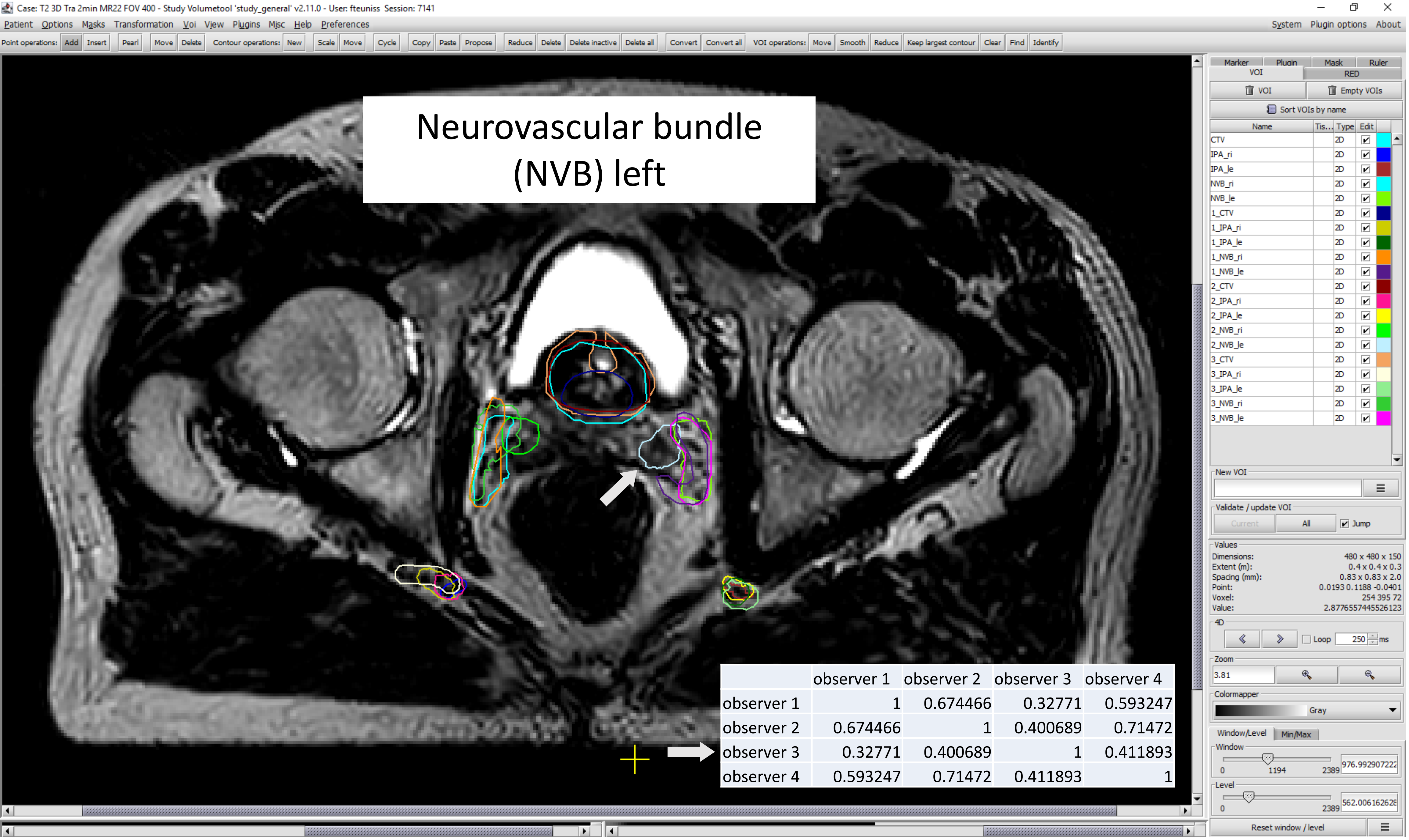

	observer 1	observer 2	observer 3	observer 4
observer 1	1	0.674466	0.32771	0.593247
observer 2	0.674466	1	0.400689	0.71472
observer 3	0.32771	0.400689	1	0.411893
observer 4	0.593247	0.71472	0.411893	1

Neurovascular bundle (NVB) left

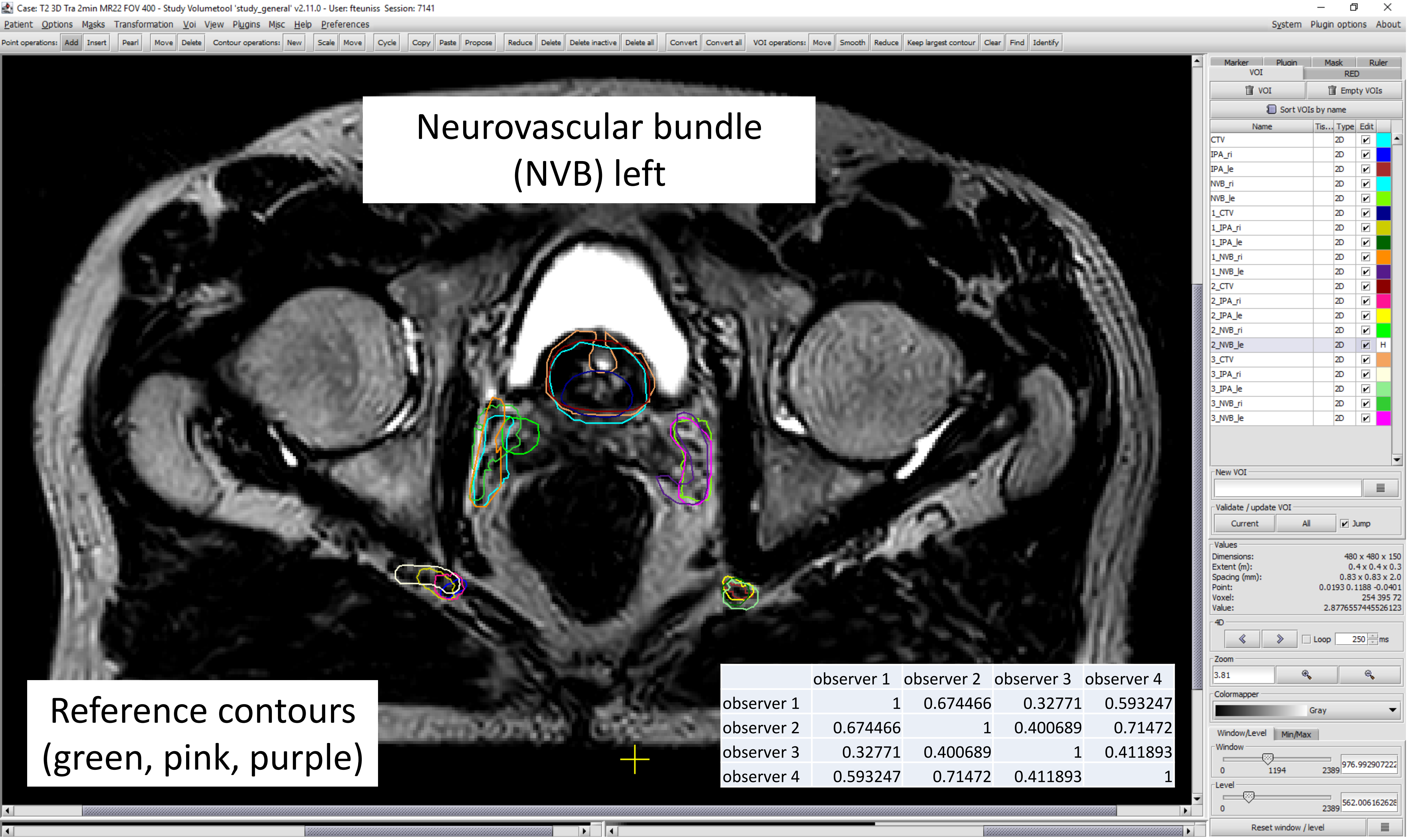

	observer 1	observer 2	observer 3	observer 4
observer 1	1	0.674466	0.32771	0.593247
observer 2	0.674466	1	0.400689	0.71472
observer 3	0.32771	0.400689	1	0.411893
observer 4	0.593247	0.71472	0.411893	1

Reference contours (green, pink, purple)

Neurovascular bundle (NVB) left

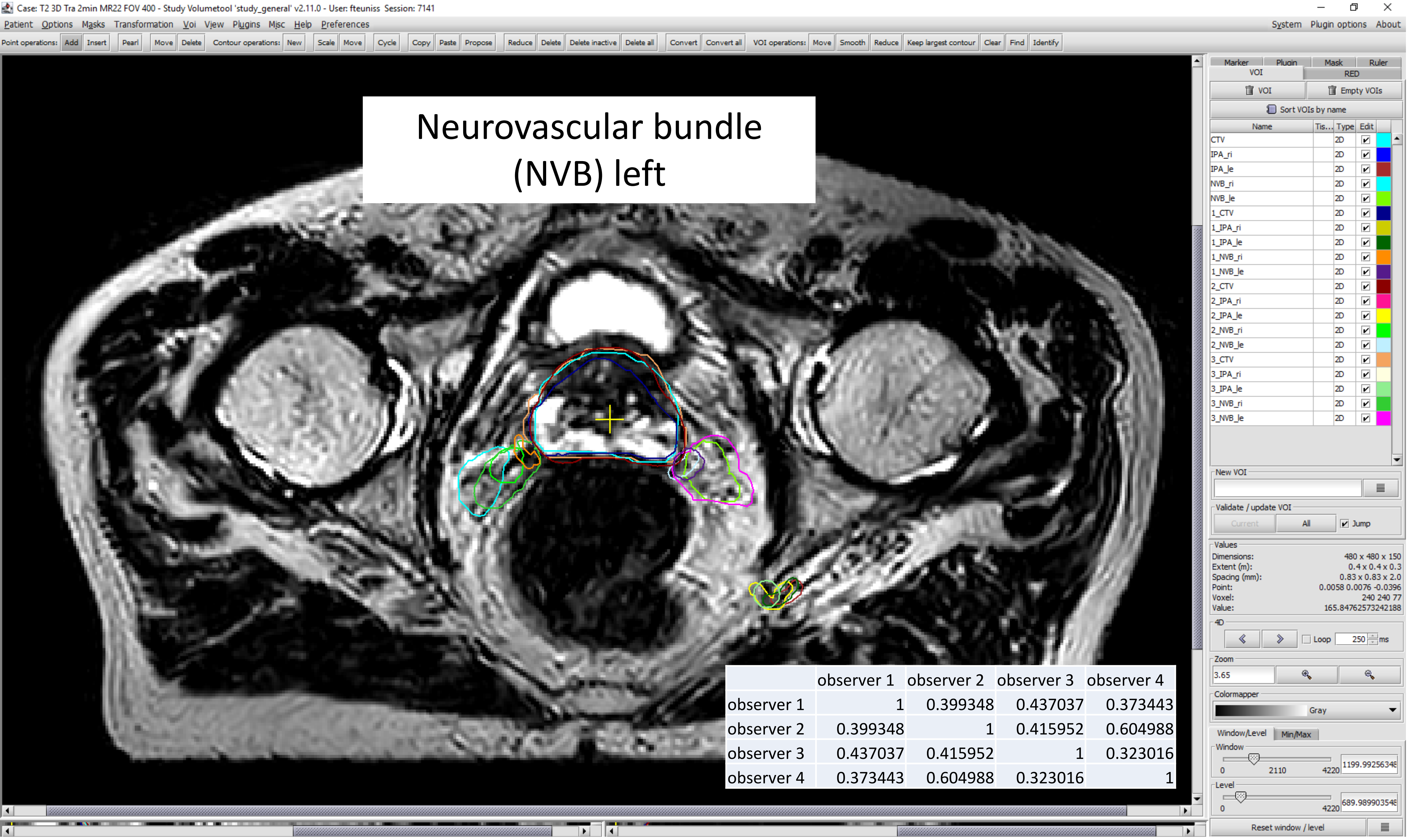

	observer 1	observer 2	observer 3	observer 4
observer 1	1	0.399348	0.437037	0.373443
observer 2	0.399348	1	0.415952	0.604988
observer 3	0.437037	0.415952	1	0.323016
observer 4	0.373443	0.604988	0.323016	1

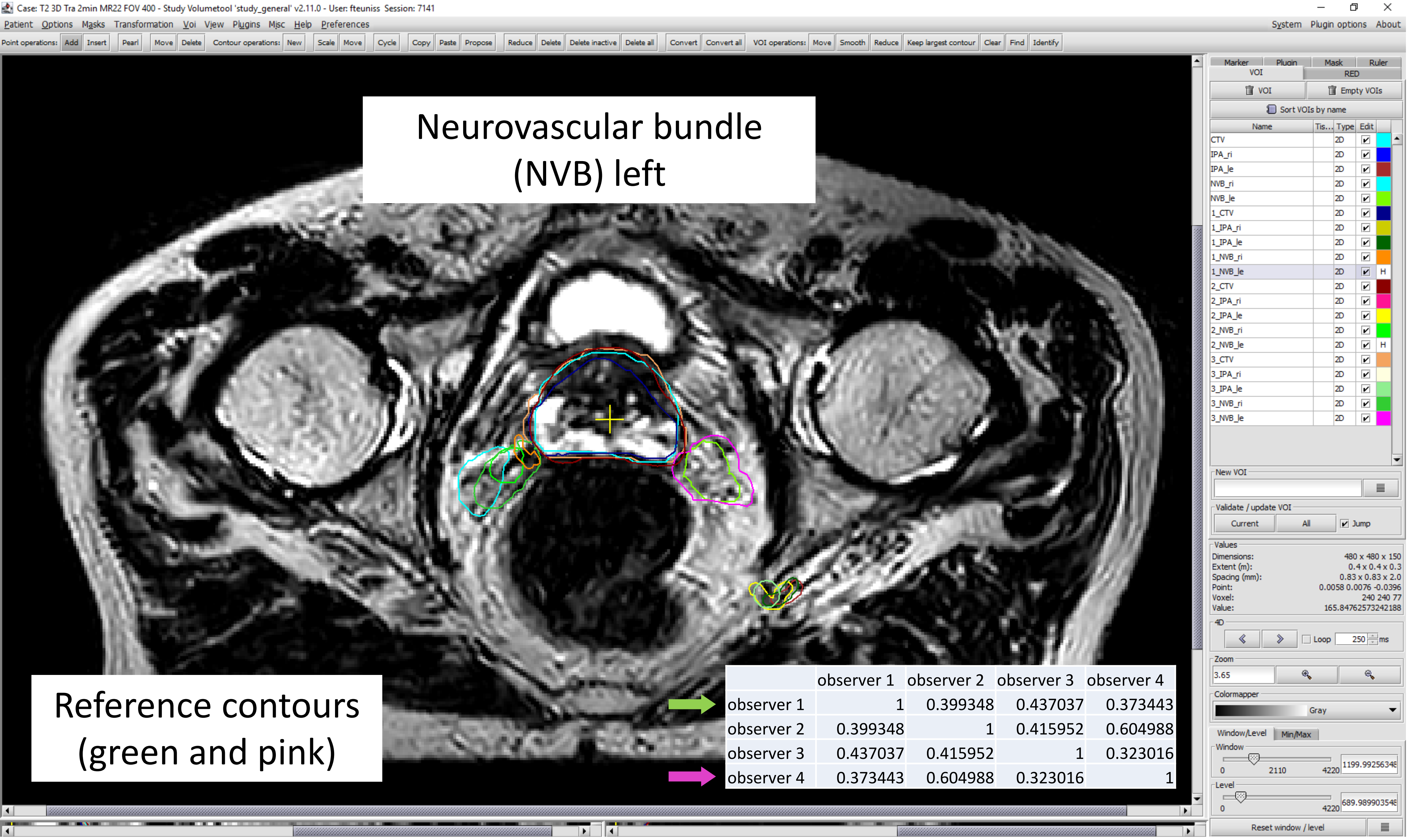

Neurovascular bundle (NVB) left

Reference contours (green and pink)

	observer 1	observer 2	observer 3	observer 4
observer 1	1	0.399348	0.437037	0.373443
observer 2	0.399348	1	0.415952	0.604988
observer 3	0.437037	0.415952	1	0.323016
observer 4	0.373443	0.604988	0.323016	1

Neurovascular bundle (NVB) left

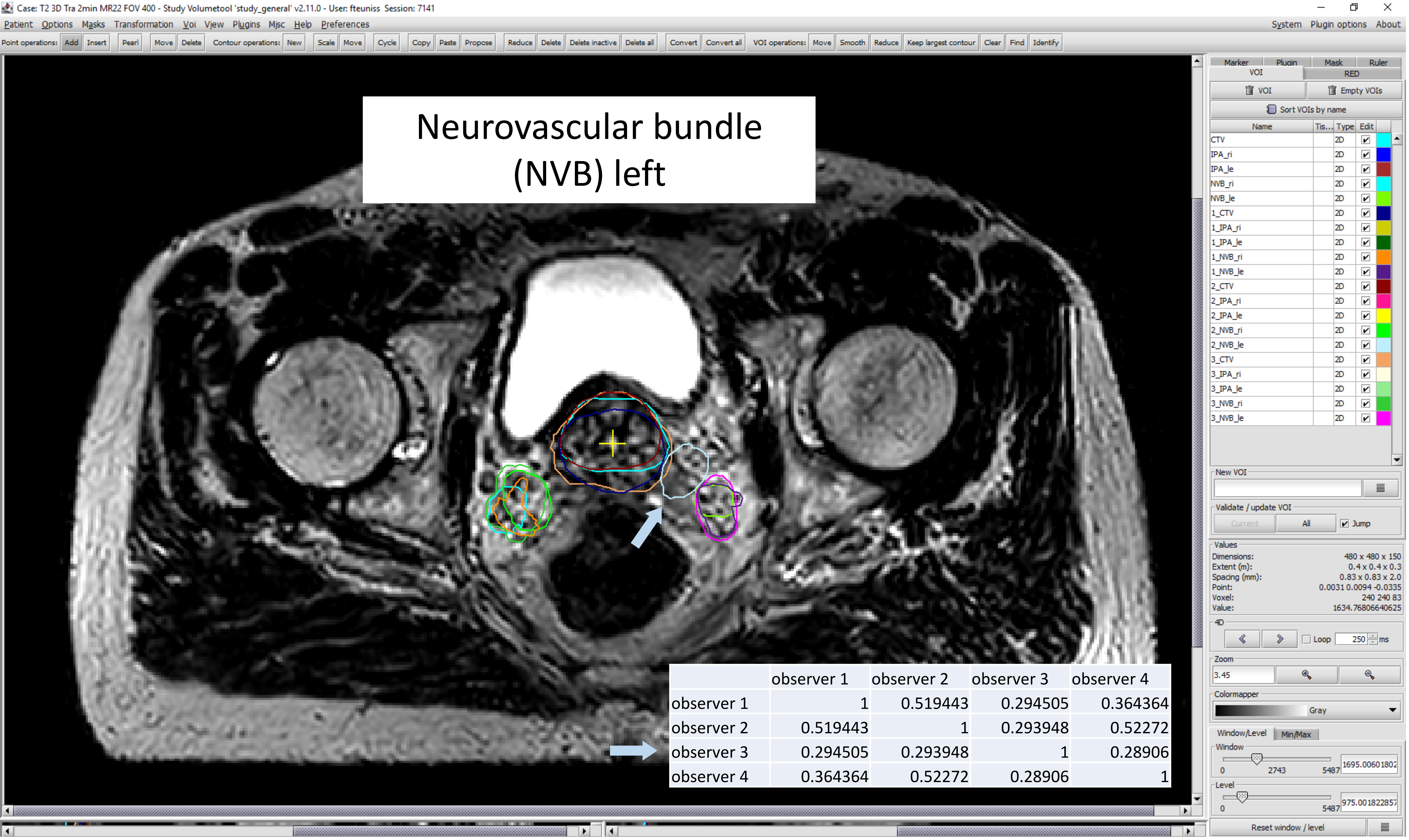

	observer 1	observer 2	observer 3	observer 4
observer 1	1	0.519443	0.294505	0.364364
observer 2	0.519443	1	0.293948	0.52272
observer 3	0.294505	0.293948	1	0.28906
observer 4	0.364364	0.52272	0.28906	1

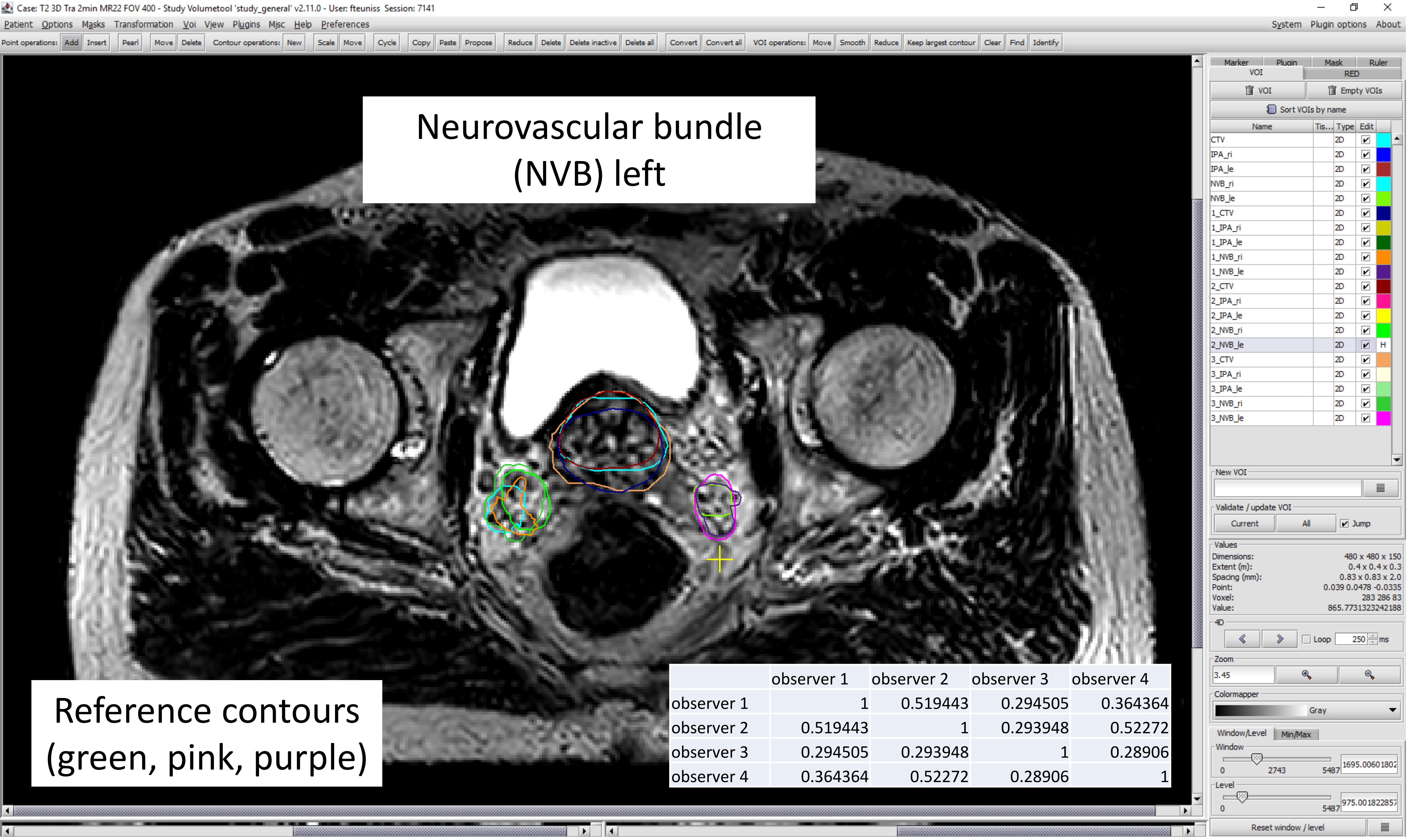

Neurovascular bundle (NVB) left

Reference contours (green, pink, purple)

	observer 1	observer 2	observer 3	observer 4
observer 1	1	0.519443	0.294505	0.364364
observer 2	0.519443	1	0.293948	0.52272
observer 3	0.294505	0.293948	1	0.28906
observer 4	0.364364	0.52272	0.28906	1

In short:

- IPA:
  - Do not contour too wide, but keep a small margin around the low signal vascular structure
- NVB:
  - Little contouring variation between observers at apex, more variation at base
  - Note: vesicles vs. NVB
  - At level of prostate base: NVB often higher signal and more lateral than vesicles
  - Contour wider at the base, narrower at the apex

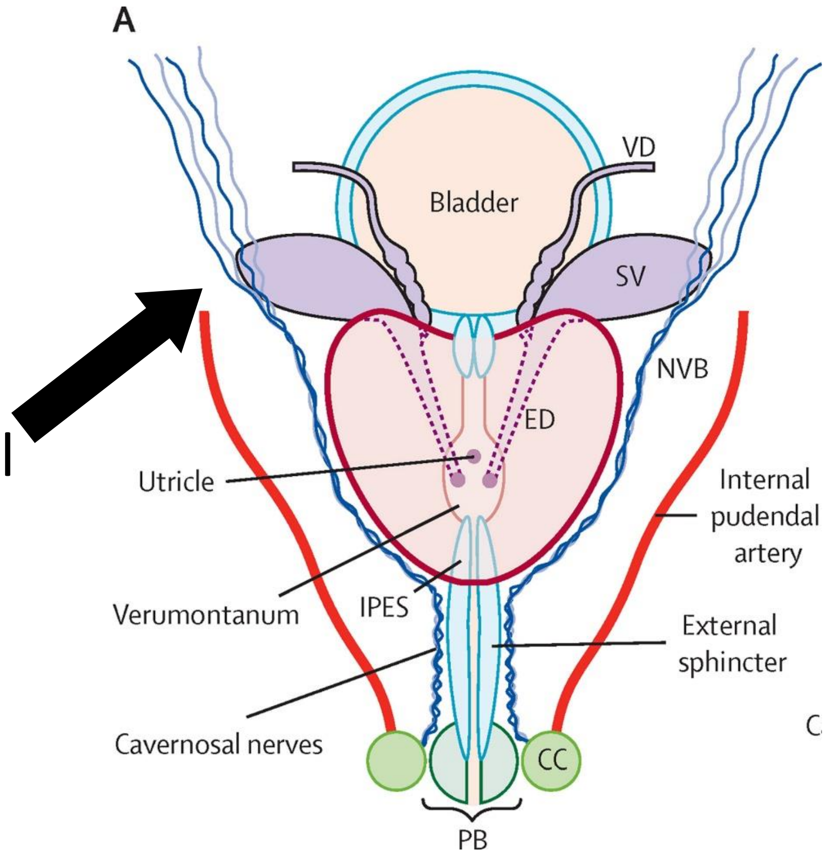

Supplement: Supplementary data 2 [file mmc2.pdf]
