# Supplementary material for: Interrater agreement of contouring of the neurovascular bundles and internal pudendal arteries in neurovascular-sparing magnetic resonance-guided radiotherapy for localized prostate cancer
Source: Clin Transl Radiat Oncol. 2021 Nov 14;32:29–34. doi: 10.1016/j.ctro.2021.11.005 (PMC8605225; doi:10.1016/j.ctro.2021.11.005)
Supplement: Supplementary data 3 [file mmc3.docx]

**Supplementary material**

Main study 3D T2-weigted TSE MRI sequence parameters.

| FOV (mm) | 400x446x180 |
| --- | --- |
| resolution (mm) | 0.78x0.78x2 |
| flipangle (degree) | 90, refocussing control: 100 |
| TE (ms) | 120 |
| TR (ms) | 1635 |
| Readout bandwidth (Hz/pixel) | 562 |

Abbreviations: TSE = turbo spin echo; FOV = field-of-view ; TE = echo time; TR = repetition time
